# Whole-Genome Sequencing Identifies a New *Aspergillus tubingensis* Strain from *Eremostachys isochila* with Intraspecific Variation in Pharmaceutically Relevant Biosynthetic Gene Clusters

**DOI:** 10.3390/molecules31213939

**Published:** 2026-11-01

**Authors:** Farkhod Eshboev, Mavjuda Kholikova, Asifullah Khan, Meixia Yang, Alex X. Gao, Kamila Mardieva, Akhror Abdurashidov, Saida Turaeva, Vera Alferova, Elvira Yusupova, Zilola Kabirova, Shokhista Tashmukhamedova, Tina T. X. Dong, Bakhodir Sagdullaev, Shaknoz Azimova, Karl W. K. Tsim

**Affiliations:** 1 Institute for Advanced Studies, New Uzbekistan University, Tashkent 100007, Uzbekistan; a.khan@newuu.uz (A.K.); a.abdurashidov@newuu.uz (A.A.); 2 Department of Biotechnology and Microbiology, National University of Uzbekistan, Tashkent 100174, Uzbekistan; mavjudaxoliqova63@gmail.com (M.K.); kabirovazilola25@gmail.com (Z.K.); shokhista.tashmukhamedova@gmail.com (S.T.); 3 Institute of Fundamental and Applied Research, National Research University TIIAME, Tashkent 100000, Uzbekistan; 4 Division of Life Science and Center for Chinese Medicine R&D, The Hong Kong University of Science and Technology, Clear Water Bay, Kowloon, Hong Kong SAR, China; mxyang@ust.hk (M.Y.); gaoxiong@ust.hk (A.X.G.); 5 S.Yu. Yunusov Institute of the Chemistry of Plant Substances, Academy of Sciences of Uzbekistan, Tashkent 100170, Uzbekistan; kamila.mardieva@gmail.com (K.M.); saidaicps@gmail.com (S.T.); kofeechek@rambler.ru (E.Y.); botina@ust.hk (T.T.X.D.); genlab_icps@yahoo.com (S.A.); 6 Shemyakin-Ovchinnikov Institute of Bioorganic Chemistry, 117997 Moscow, Russia; alferovava@gmail.com; 7 A.S. Sadykov Institute of Bioorganic Chemistry, Academy of Sciences of Uzbekistan, Tashkent 100143, Uzbekistan; bsagdullaev29@gmail.com

**Keywords:** fungal endophyte, biosynthetic gene cluster (BGC), secondary metabolite biosynthesis, *Aspergillus tubingensis* S23, comparative genomics

## Abstract

Fungal endophytes are promising sources of pharmaceutically relevant secondary metabolites, yet the genomic basis of their biosynthetic potential remains poorly characterized. We sequenced the genome of endophyte S23, isolated from healthy *Eremostachys isochila*, and compared it with 13 reference genomes. ITS, multilocus, and genome-scale phylogenies identified S23 as *Aspergillus tubingensis*. CAZyme, secretome, effector, and virulence-factor analyses showed limited plant-pathogenic potential, with secreted LysM-domain proteins, chitinases, and β-1,3-glucanases as candidate determinants of endophytic adaptation. Biosynthetic gene cluster (BGC) mining identified 95 clusters, mostly conserved across *A. tubingensis*, including one S23-specific type I polyketide synthase cluster. S23 was identified as the only *A. tubingensis* genome carrying the complete ten-gene yanuthone D cluster, while the other five strains retained only three cluster genes and lacked the core polyketide synthase gene. This locus conflicts with the species phylogeny, arguing against simple vertical inheritance. The burnettiene A cluster showed a patchy intraspecific distribution, being complete in S23 but absent from three *A. tubingensis* strains. Nidulanin A and TAN-1612 BGCs were conserved, and the core kojic acid gene, *kojA*, was absent from all *A. tubingensis* genomes, including S23. These findings highlight distinctive biosynthetic features of S23 and provide targets for experimental validation of its metabolite production.

## 1. Introduction

Fungal endophytes, which colonize internal plant tissue, are recognized as a major reservoir of chemically diverse and biomedically relevant secondary metabolites. Numerous bioactive compounds have been reported from cultivable endophytic fungi over the past three decades [1]. However, the relationship between phenotypic bioactivity reports and their underlying genetic basis has historically been addressed for only a small fraction of characterized endophyte isolates. The majority of endophyte natural-product discovery has proceeded through chemical isolation and bioassay alone, without a corresponding genomic or biosynthetic gene cluster (BGC) analysis [2]. Genome sequencing and genome mining address this gap, enabling systematic detection of an isolate’s full biosynthetic gene cluster repertoire, including which genetic loci are responsible and whether they are conserved or isolate-specific within the lineage. These approaches also unveil the cryptic or silent pathways that remain undetected by chemical screening alone [3].

The genus *Aspergillus* is a particularly well-established source of industrially and pharmaceutically significant secondary metabolites, reflecting a large and biologically diverse biosynthetic gene cluster repertoire across the genus [4,5]. The section Nigri (the “black aspergilli”) is of particular economic importance within this genus. *Aspergillus niger* alone accounts for approximately 90% of the world’s industrial citric acid production [6]. Other section Nigri species are used directly in traditional food and beverage fermentation across East Asia [5]. At the same time, section Nigri remains taxonomically complex, and species boundaries within the section have been repeatedly revised as molecular phylogenetic methods have advanced. The ITS barcoding alone is insufficient to resolve species-level identity within the section, and recent studies have proposed conflicting synonymy schemes for closely related species pairs [7,8,9,10,11].

Beyond industrial enzyme and organic acid production, several species in the section Nigri and related *Aspergillus* species are reported producers of structurally diverse, pharmacologically active secondary metabolites. These span meroterpenoids with antifungal activity, cytotoxic polyketides, anthraquinone-type neuropeptide receptor antagonists, and pyranone-class enzyme inhibitors already reported to have commercial cosmetic and pharmaceutical use [12,13,14]. Genome-wide biosynthetic gene cluster mining provides a systematic route to detect genomic evidence for these pathways in a new isolate. Cross-referencing this against curated, experimentally characterized reference databases, such as MIBiG, provides further validation. This approach also allows isolate-specific biosynthetic novelty to be distinguished from features that are simply conserved, ancestral characteristics of the broader taxonomic lineage [15,16].

Endophytic fungi occupy an ecological continuum between mutualism and latent pathogenicity [17]. Their genomes typically encode a mixture of plant cell-wall-degrading enzymes (CAZymes), secreted effector proteins [18,19], and other virulence-associated factors. The presence of these features alone does not resolve whether a given isolate behaves as a pathogen, a neutral commensal, or a protective mutualist toward its host [17]. Comparative genomics against a reference panel of close relatives, along with screening against curated, experimentally validated virulence factor and effector databases, offers a systematic, evidence-based approach to explore such ecological positioning directly from genomic content [20,21].

In this study, we present the whole-genome characterization of isolate S23, a filamentous fungal endophyte recovered from healthy, asymptomatic tissue of *Eremostachys isochila* (Lamiaceae). We address the precise taxonomic identity and phylogenetic placement of isolate S23 via ITS barcoding, multi-locus protein-coding phylogenetics, and genome-scale ortholog-based phylogenomics. The isolate’s likely mode of ecological interaction with host plant was assessed through comparative CAZyme profiling, secretome/effector prediction [22,23], and curated virulence-factor database screening [24] against a 13-genome reference panel of close relatives. The isolate’s pharmaceutically or industrially relevant secondary metabolite potential was investigated through genome-wide biosynthetic gene cluster detection. Furthermore, the comparative and evolutionary basis of pharmaceutically or industrially promising BGCs was investigated.

## 2. Results

### 2.1. Whole Genome Sequencing, Assembly and Annotation

Whole-genome sequencing generated 2 × 150 bp paired-end reads for the isolate S23. Low-quality bases, adapter sequences, and other low-quality reads were filtered out using fastp [25]. The draft genome assembly of isolate S23 comprised 155 contigs with a total length of 35,631,391 bp (35.63 Mb) and a largest contig of 1,791,809 bp. The *de novo* assembly had an N50 of 412,769 bp, N90 of 117,932 bp, L50 of 25, and GC content of 48.97%. SPAdes did not join any contigs into gapped scaffolds, so the assembly contains no N-gaps. The mean sequencing depth was approximately 169× (about 20.05 million 2 × 150-bp read pairs; Section 4.3). Coverage was uniform across most of the assembly, and GC content was unimodal for 147 of 155 contigs (~35.4 Mb), with a genome-wide mean of 48.97%. The remaining eight contigs of 223.9 kb, which constitute 0.63% of the total assembly, were investigated individually. Four short contigs (8.96 kb combined) showed normal nuclear-range GC (51–59%) with 20- to 100-fold elevated SPAdes k-mer coverage (488–2059×, compared with a genome-wide median of ~21×), which is consistent with multi-copy ribosomal DNA repeats. Of the remaining four low-GC contigs, two were mitochondrial, with 25–26% GC and elevated coverage. One was identified by BLASTn (BLAST+ v2.17.0) as the mitochondrial genome (99.99% identity and 100% coverage to *Aspergillus tubingensis* WU-2223L mitochondrial DNA), and the other was a redundant assembly of the same circular mitogenome. The other two low-GC contigs had no significant GenBank match, showing only weak similarity to *Aspergillus* sequences and no sign of non-fungal origin. Together, these results indicate a single fungal genome without contamination, with the atypical contigs derived from mitochondrial and rDNA sequences.

BUSCO assessment indicated 99.0% genome completeness (98.9% single-copy, 0.1% duplicated), with 0.6% fragmented and 0.4% missing BUSCOs, indicating a near-complete, high-quality draft assembly consistent in size and GC content with genomes of *Aspergillus* section Nigri. Ab initio gene prediction with AUGUSTUS identified 10,728 protein-coding genes for the S23 genome assembly, with a mean predicted protein length of 527 amino acids (range: 33 to 6210 aa). This is consistent with the expected gene complement of an *Aspergillus* genome. Because these gene models are ab initio predictions, gene presence, absence, or disruption in the key biosynthetic gene clusters was verified directly against the genomic sequences.

### 2.2. ITS and Multi-Locus Sequence-Based Taxonomic Characterization

BLASTn analysis of the partial ITS sequence placed isolate S23 within *Aspergillus* section Nigri, with top hits to *A. niger* (99.24% identity, 100% query coverage) and closely related section Nigri species, including *A. welwitschiae*, *A. tubingensis*, *A. foetidus*, *A. awamori*, *A. phoenicis*, *A. costaricensis*, and *A. piperis*, at 98.98% identity (Table S1). This result confirms only the section-level placement of isolate S23, because ITS does not reliably resolve the species-level relationships within section Nigri [7]. A definitive species assignment for isolate S23 therefore needs additional multi-locus analysis.

In contrast to the provisional ITS-based placement, the three independent protein-coding loci (benA, calmodulin gene (CaM), and RPB2) unambiguously identified the isolate S23 as *Aspergillus tubingensis*. The top BLASTn hit for benA was *A. tubingensis* (100.00% identity, 99% coverage), while the *A. niger* reference hits were notably lower (94.21% identity; Table S2). Likewise, the CaM BLASTn hits were dominated by *A. tubingensis* with 100.00% identity, and other section Nigri species appearing only at low identity (Table S3). *A. tubingensis* was detected as the top hit for RPB2 BLASTn as well, with 99.94% identity and 100% coverage, clearly separated from low *A. niger* identity (94.88% identity; Table S4). The convergence of all these independent marker loci on *A. tubingensis* confirms the known limitation of ITS in resolving species boundaries within this section [7,8].

**Figure 1:**
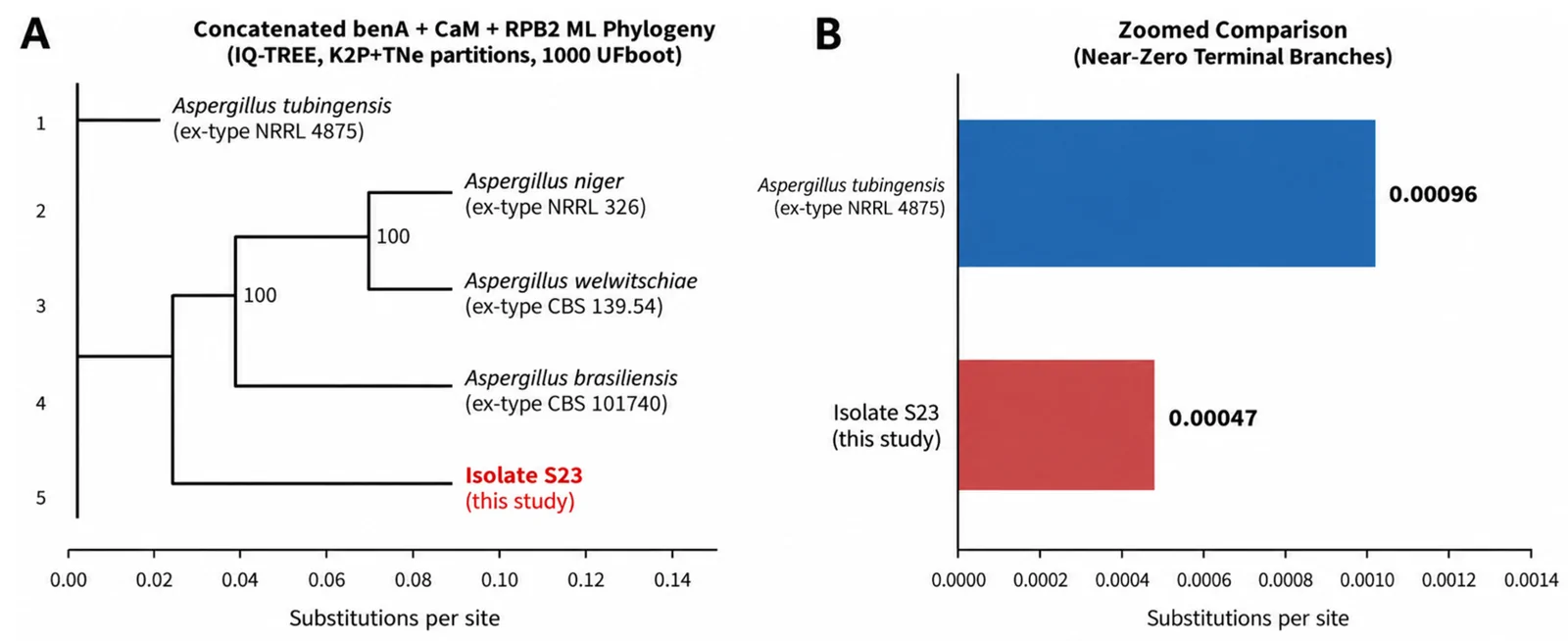
Maximum-likelihood phylogenetic analysis of *A. tubingensis* S23 based on concatenated benA, CaM, and RPB2 sequences. (**A**) Maximum-likelihood phylogenetic tree constructed using IQ-TREE with the K2P+TNe substitution model and 1000 ultrafast bootstrap replicates. Branch lengths represent substitutions per site, and node labels indicate ultrafast bootstrap support values (%). The tree is unrooted. (**B**) Enlarged comparison of the near-zero terminal branch lengths of isolate S23 (0.00047 substitutions per site) and the *A. tubingensis* ex-type strain NRRL 4875 (0.00096 substitutions per site).

A maximum-likelihood phylogeny was constructed from concatenated benA, CaM, and RPB2 sequences (2568 bp), using K2P + I, K2P, and TNe as the best-fit partition models. In this unrooted five-taxon tree, isolate S23 and the ex-type strain of *A. tubingensis* (NRRL 4875) grouped together, separated from the *A. niger*–*A. welwitschiae* pair and *A. brasiliensis* by a branch with 100% ultrafast bootstrap support (Figure 1). Consistent with this topology, the patristic distance between S23 and *A. tubingensis* NRRL 4875 was 0.0014 substitutions/site, which is about 50-fold smaller than the distances from S23 to the other species (≥0.07 substitutions/site). The species assignment therefore rests on this topology, together with the genome-scale phylogeny (Section 2.3), and not on terminal branch lengths, which are given for completeness only (S23, 0.00047; *A. tubingensis*, 0.00096; *A. niger*, 0.00751; *A. welwitschiae*, 0.00438; *A. brasiliensis*, 0.04733 substitutions/site).

### 2.3. Genome-Scale Phylogenomics

**Figure 2:**
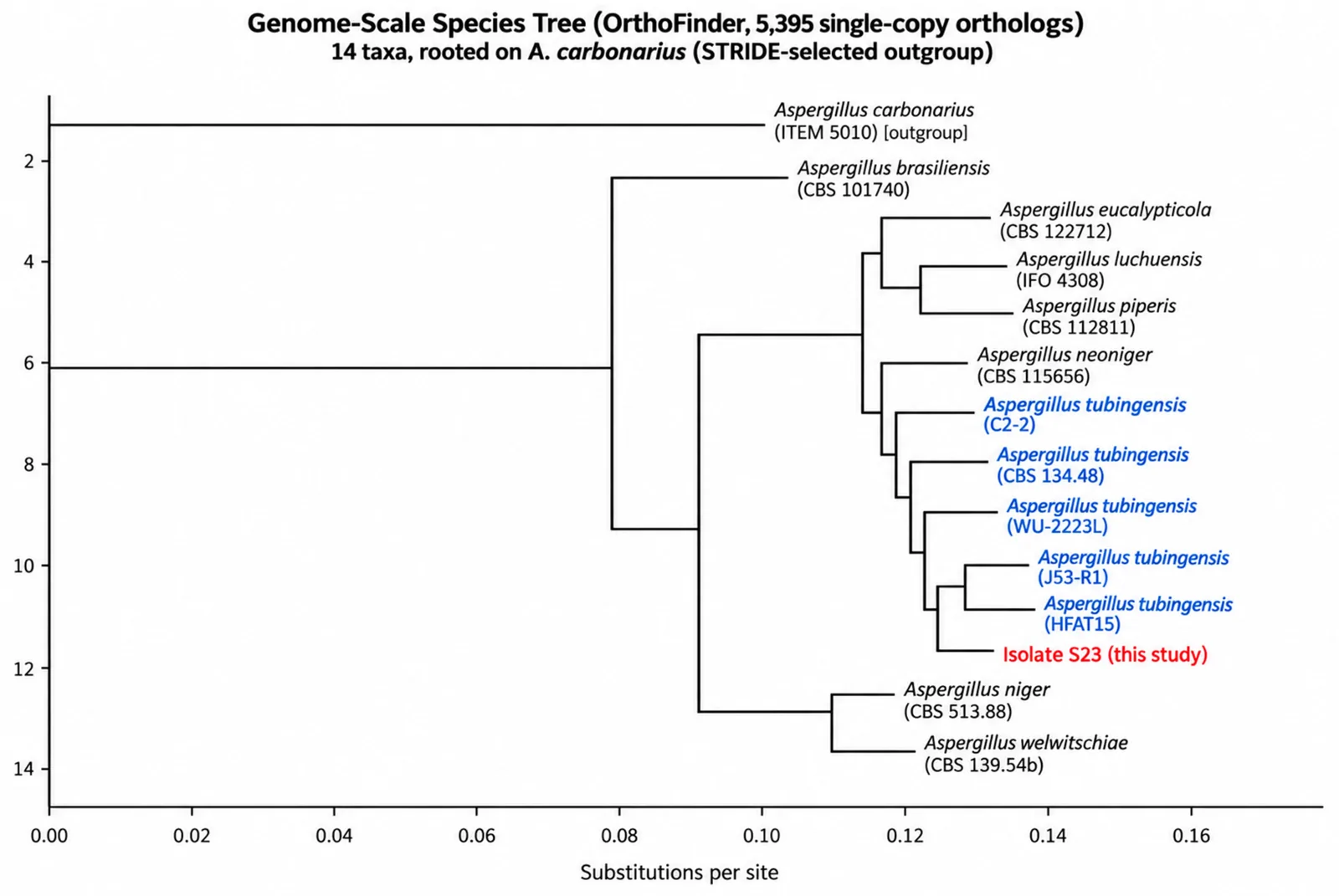
Genome-scale species tree based on 5395 single-copy orthologs from 14 taxa, rooted on *Aspergillus carbonarius*.

In addition to individual marker loci, genome-scale orthology analysis was pursued to assess broader and statistically more robust phylogenetic relationships among isolate S23 and its closest relatives. OrthoFinder analysis grouped 96.3% of 160,982 genes from isolate S23 and 13 reference genomes into 12,998 orthogroups, of which 5395 were single-copy in every genome. The species tree based on these 5395 single-copy ortholog sequences fully resolved isolate S23 within the *A. tubingensis* clade, placing it as sister to a clade comprising the JS3-R1 and HFAT15 strains (Figure 2). S23 is therefore nested within *A. tubingensis*, and its short terminal branch (0.00273 substitutions/site) indicates that it does not represent a divergent lineage. *Aspergillus carbonarius*, chosen as the outgroup on the basis of previous section Nigri phylogenies [9], was also identified as the root by OrthoFinder’s STRIDE algorithm.

### 2.4. CAZyme, Secretome, and Effector Profiles

**Figure 3:**
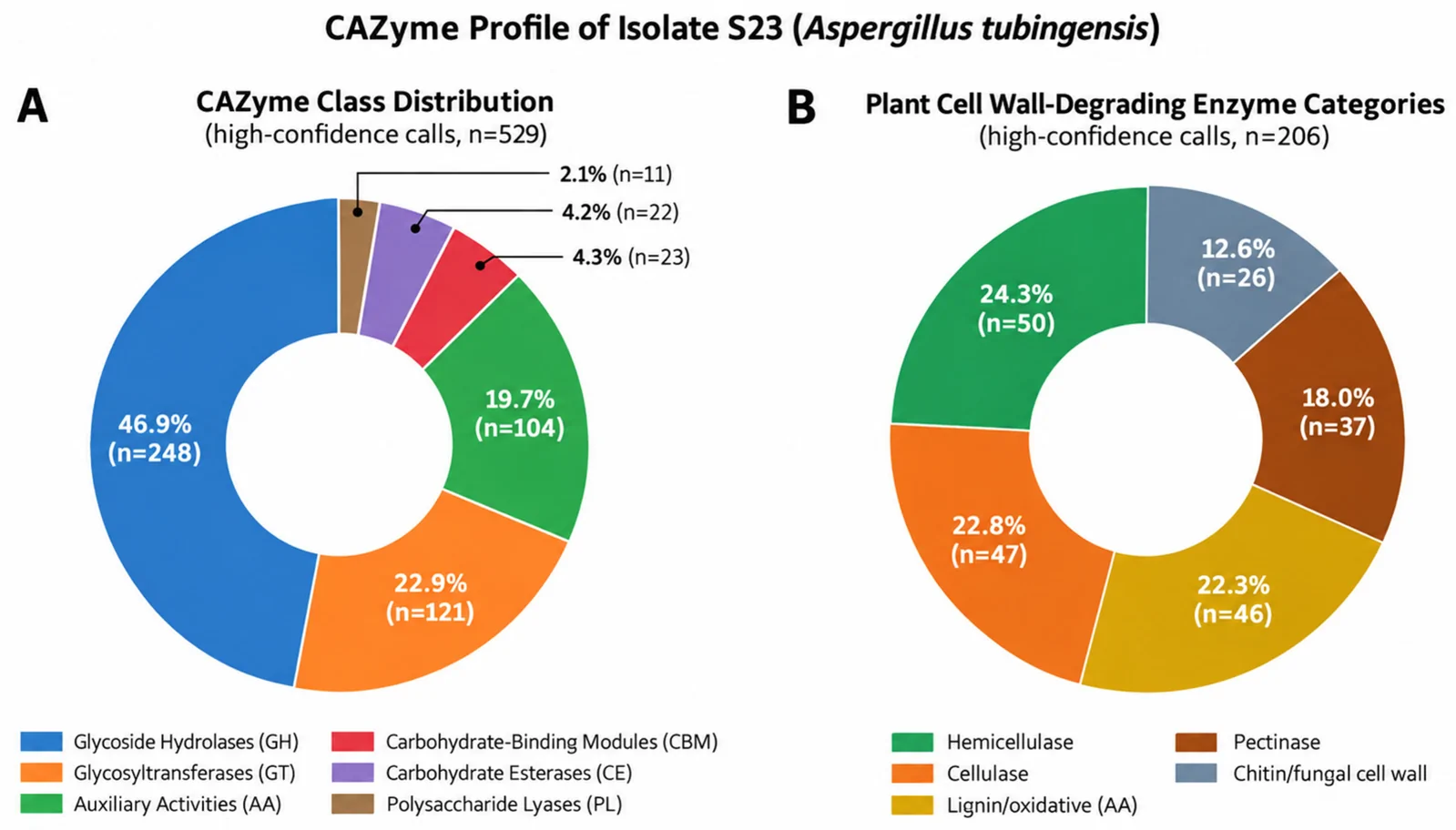
High-confidence CAZyme profile of isolate S23 (*Aspergillus tubingensis*): (**A**) Distribution across the six major CAZyme classes. (**B**) Distribution across plant cell wall-degrading enzyme categories.

Carbohydrate-active enzymes (CAZyme) content reflects a fungal isolate’s capacity for plant biomass interaction, which is relevant to both its ecological role and industrial potential as an endophyte. Out of 10,728 predicted proteins of the S23 isolate, 2145 (20.0%) carried at least one detectable CAZyme domain. Among these, 529 (24.7%) were supported by at least two of the three annotation methods as a high-confidence set (Supplementary Data S1). These high-confidence CAZymes spanned all six major classes, including glycoside hydrolases (GH; n = 248, 46.9%), glycosyltransferases (GT; n = 121, 22.9%), auxiliary activities (AA; n = 104, 19.7%), carbohydrate-binding modules (CBM; n = 23, 4.3%), carbohydrate esterases (CE; n = 22, 4.2%), and polysaccharide lyases (PL; n = 11, 2.1%) (Figure 3A). Among the 529 high-confidence CAZyme set, 206 genes were classified into different categories of plant cell wall-degrading enzymes. These are hemicellulases (n = 50, 24.3%), cellulases (n = 47, 22.8%), lignin-active/oxidative enzymes (AA classes; n = 46, 22.3%), pectinases (n = 37, 18.0%), and chitin/fungal cell wall active enzymes (n = 26, 12.6%) [26,27] (Figure 3B). The substrate prediction (dbCAN-sub) scan supported a broad, balanced plant polysaccharide degradation capacity of isolate S23, with pectin, host glycan, β-glucan, lignin, chitin, α-glucan, xylan, and arabinan as the top substrates. This CAZyme repertoire comprises approximately one-fifth of the predicted proteome of isolate S23, which is congruent with the substantial biomass degradation capability of section Nigri fungi [28].

The secreted proteome represents the molecular interface of a fungus with its host. SignalP 6.0 predicted 919 secreted proteins (8.6% of the proteome; Supplementary Data S2). Of these, 235 (25.6% of the secretome) were classified as candidate effectors by EffectorP-fungi 3.0, comprising 135 apoplastic, 57 cytoplasmic, and 43 dual-localization (apoplastic/cytoplasmic) candidates. The predominance of apoplastic over cytoplasmic candidates suggests limited potential for invasive, host cell penetrating colonization of S23.

Because section *Nigri* includes mycotoxin producers and opportunistic pathogens [29], the secretome was therefore screened against curated virulence-factor databases (PHI-base and DFVF) as confirmatory evidence. Among the 919 predicted secreted proteins, 434 (47.2%) exhibited significant similarity to proteins documented in the PHI-base database (Supplementary Data S3). For 151 of these 434 matches (34.8%), the PHI-base mutant phenotype was “unaffected pathogenicity”. Among the 113 closest matches (≥50% identity), the proportion was similar (i.e., 38; 33.6%). This indicates that a PHI-base match does not by itself identify a virulence factor, because about one third of the matched genes did not affect pathogenicity when mutated in the original pathogen. This pattern points to broadly conserved housekeeping and cell wall-related genes rather than a specialized virulence repertoire. Only six secreted proteins matched experimentally confirmed plant-pathogen effectors at ≥50% identity (Table S5).

The high-confidence CAZyme set contained 15 GH18 chitinase and 14 GH16 β-1,3-glucanase genes, of which 17 are secreted and have PHI-base matches (Supplementary Data S3). This enzyme combination resembles the fungal cell wall degradation strategy of fungal biocontrol agents such as *Trichoderma* species [26,27], and is therefore presented as a candidate antagonistic mechanism to be tested experimentally. LysM-domain proteins, which allow fungi to evade chitin-triggered host immunity, were identified in 41 genes (Pfam PF01476 or CBM50), 17 of which are predicted to be secreted (Table S6). Five of these proteins (g694.t1, g2556.t1, g4539.t1, g5323.t1 and g9285.t1) match Ecp6, a confirmed LysM effector of *Fulvia fulva* [18], in PHI-base (32–37% identity). All five are also predicted by EffectorP to be apoplastic effectors. Because the same chitin-evasion mechanism operates in both pathogenic fungi and mutualistic root endophytes [18,19], these genes are candidate, rather than diagnostic, determinants of the interaction of S23 with its host.

### 2.5. Biosynthetic Gene Cluster Content and Gene Cluster Family (GCF) Network Analysis

**Figure 4:**
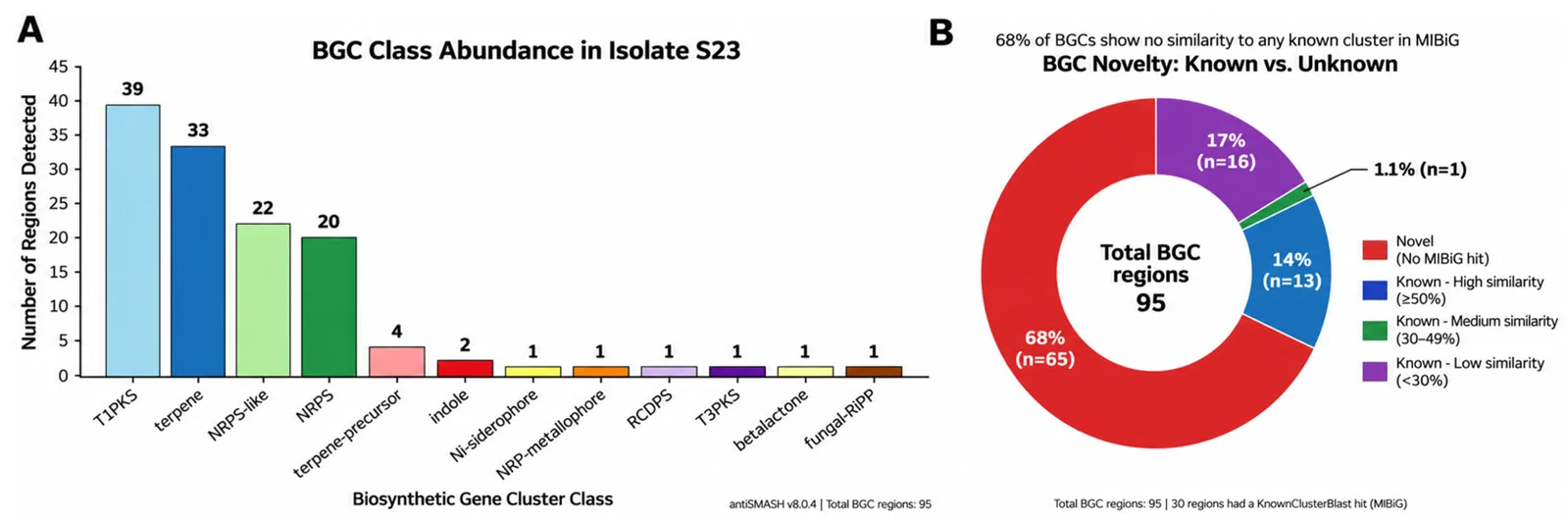
Overview of isolate S23’s biosynthetic gene cluster (BGC) content. (**A**) BGC class abundance, as detected by antiSMASH v8.0.4 (n = 95 total BGC regions). Because 26 of the 95 regions are hybrid clusters assigned to more than one class, the class counts sum to 126 class assignments rather than 95 regions. (**B**) Isolate S23’s biosynthetic gene clusters relative to the MIBiG-characterized clusters, based on similarity to KnownClusterBlast hits.

antiSMASH identified 95 biosynthetic gene clusters (BGCs) for the genome assembly of isolate S23 (Supplementary Data S4). These are dominated by type I polyketide synthase (T1PKS, n = 39) and terpene (n = 33) clusters, followed by NRPS-like (n = 22) and NRPS (n = 20) clusters, and smaller numbers of terpene-precursor, indole, and other cluster types (Figure 4A). Among these, 30 BGC regions were identified to have a KnownClusterBlast hit against MIBiG (Supplementary Data S5), while the remaining 65 (68.4%) showed no hit. Among the 30 matched regions, 13 (13.7% of all 95 regions) showed high similarity (≥50%), 1 (1.1%) medium similarity (30–49%), and 16 (16.8%) low similarity (<30%), based on each region’s highest-similarity hit (Figure 4B).

Ten BGCs of isolate S23 matched with 100% similarity to the MIBiG reference cluster. Among these, the YWA1/naphthopyrone melanin biosynthesis pathway was identified in two separate genomic regions. Its presence is consistent with black conidial pigmentation, a broadly characteristic trait of section Nigri. The remaining 100% similarity matches were burnettiene A, sesterfisherol, clavaric acid [30], TAN-1612, AbT1, choline, 6-methylsalicylic acid, and a fungisporin-type cyclic tetrapeptide family (cyclo-(D-Phe-L-Phe-D-Val-L-Val), with nine additional structural variants from the same cluster BGC0000357). These fungisporin-type cyclic peptides are hydrophobic NRPS products first characterized in *Penicillium* [31]. A separate cluster showing homology to the kojic acid biosynthetic pathway was identified at 66% similarity. Several BGCs annotated for isolate S23 show similarity to MIBiG reference clusters encoding compounds with reported pharmaceutical, cosmetic, or antimicrobial activity (Table 1). These activities were reported for the reference compounds, and their production by S23 remains to be tested.

BiG-SCAPE network analysis grouped 91 of 95 S23 BGCs (95.8%) into families containing no MIBiG reference cluster. This stricter criterion confirms that most S23 BGCs lack closely related characterized references (Supplementary Data S6); we therefore describe them as uncharacterized rather than novel. Comparison against the 13-genome panel identified two gene cluster families found only in S23. These are NODE_27 region 1 (T1PKS) and NODE_2 region 6 (NRPS–terpene). Because family-level absence can also result from annotation or clustering differences, every protein encoded in both regions was searched individually (tBLASTn (BLAST+ v2.17.0)) against the raw genome sequence of each panel genome, and the core enzymes were additionally searched against each proteome (BLASTP (BLAST+ v2.17.0); Table S7). Most accessory genes of both regions were detected in all panel genomes. In contrast, the 2214-amino-acid core polyketide synthase of the NODE_27 region had no ortholog in any panel genome. The best hits (29–39% identity) for this core gene were distant paralogs, identifying this region as an S23-specific BGC. In every panel genome, most accessory genes of this region were co-located on a single contig, whereas the main core-PKS hit was located on a different contig, indicating that the corresponding locus in these genomes lacks the polyketide synthase. The 2841-amino-acid core NRPS of the NODE_2 region was not unique to S23. A divergent homolog (75–81% amino-acid identity) was present in *A. tubingensis* CBS 134.48 and HFAT15, *A. luchuensis*, *A. neoniger*, and *A. piperis*, and a more distant one (65%) in *A. niger*. The remaining genomes carried only distant paralogs (40–50%). This region is therefore a divergent variant rather than an S23-specific cluster.

**Figure 5:**
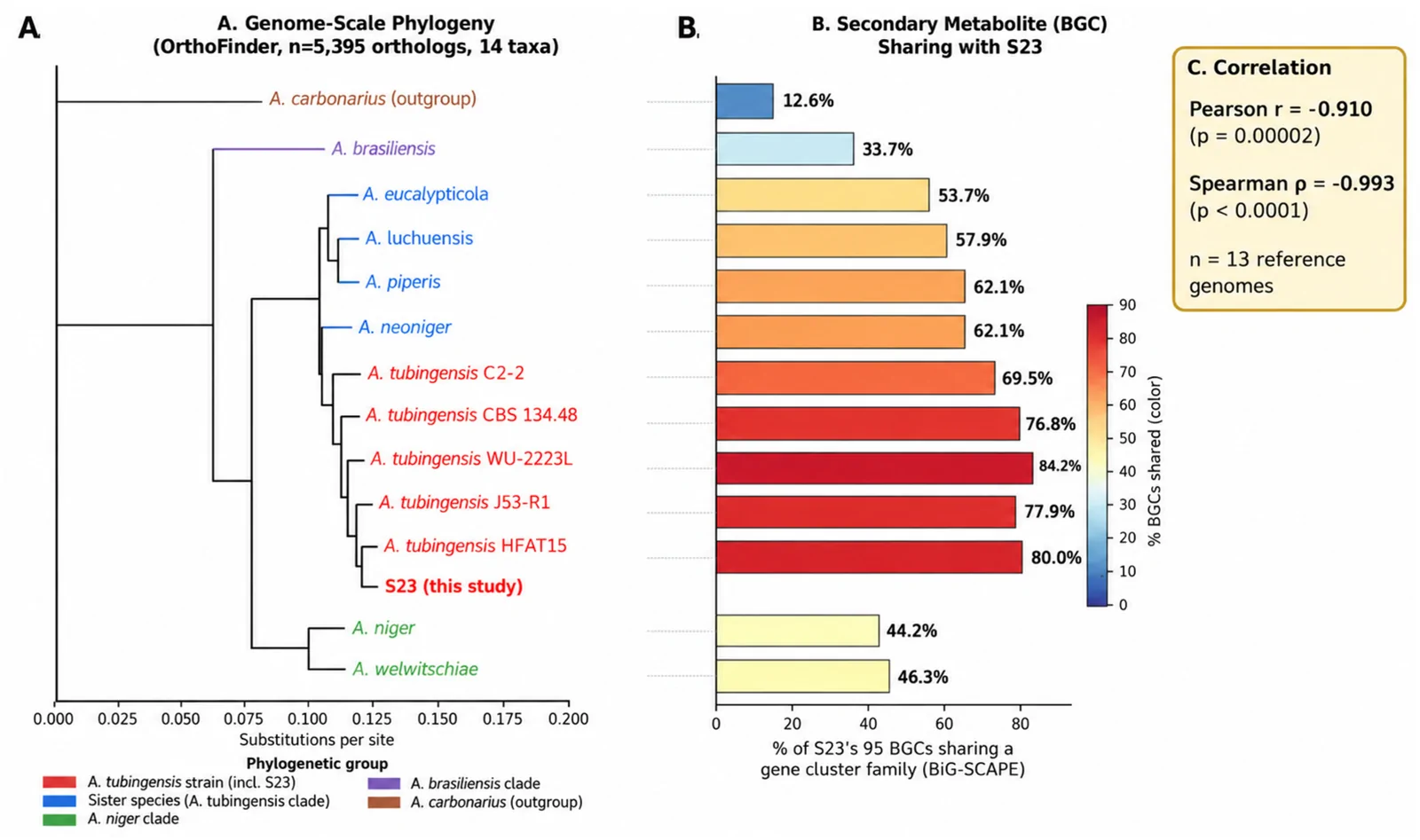
Congruence between genome-scale phylogenetic distance and biosynthetic gene cluster sharing trend. (**A**) Genome-scale phylogeny (OrthoFinder). (**B**) Isolate S23’s BGCs sharing percentage of a gene cluster family with each reference genome. (**C**) Correlation statistics.

The BGC sharing proportion of isolate S23 across the panel genomes, followed the pattern of genome-scale phylogenetic distance and BGCs sharing was highest with the most closely related strain, *A. tubingensis* WU-2223L (i.e., 84.2% of BGC families), and lowest with the outgroup, *A. carbonarius* (i.e., 12.6%) (Pearson r = −0.910, *p* = 0.00002; Spearman ρ = −0.993, *p* < 0.0001; Figure 5; Figures S1 and S2; Supplementary Data S7 and S8). This pattern independently supports the phylogenetic placement of isolate S23 using secondary metabolite genes rather than single-copy orthologs.

#### 2.5.1. Yanuthone D Biosynthetic Gene Cluster: Comparative and Evolutionary Analysis

**Figure 6:**
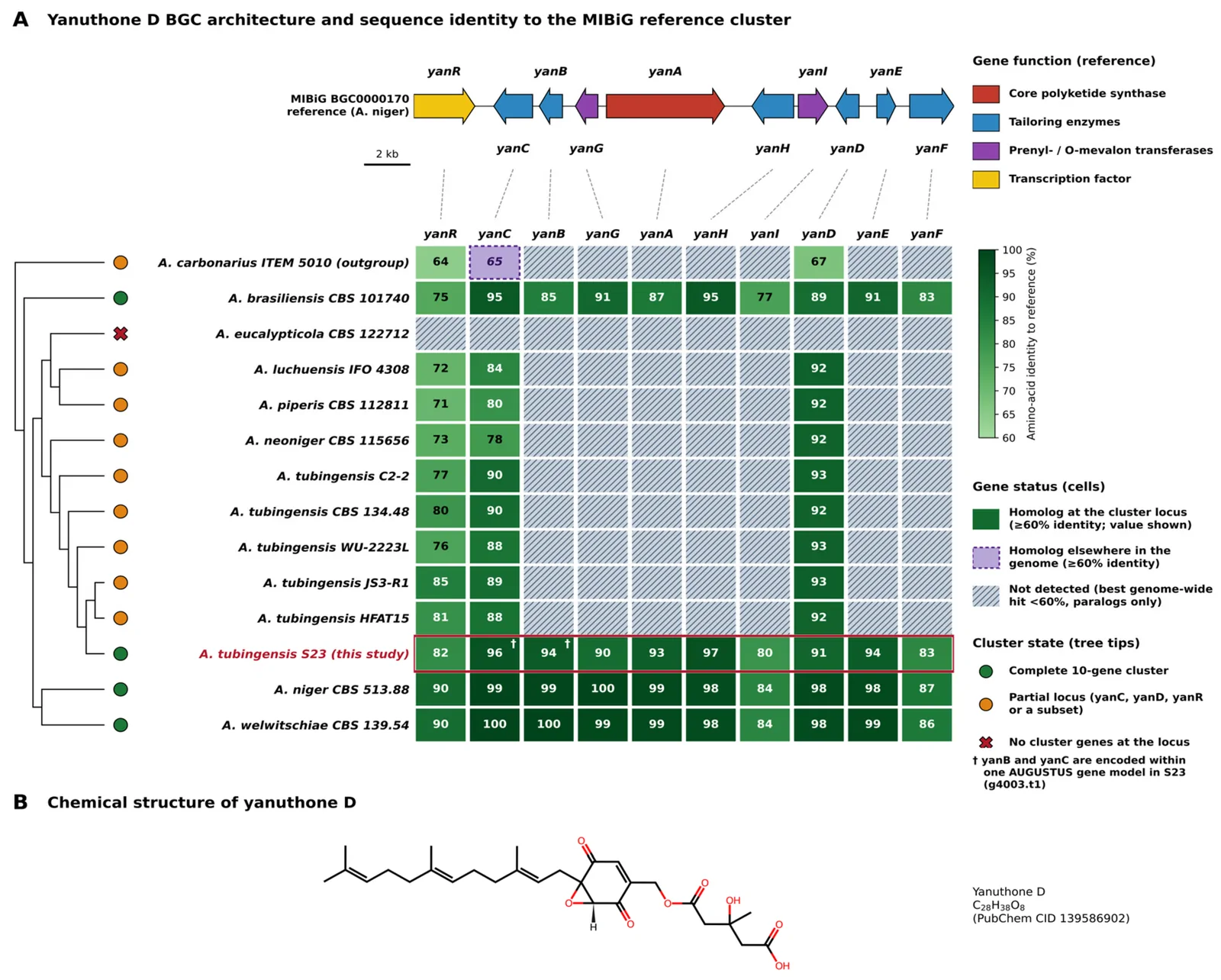
Distribution of the yanuthone D biosynthetic gene cluster (BGC) across the 14-genome panel. (**A**) Yanuthone D BGC architecture and sequence identity to the reference cluster defined by Holm et al. [12] (MIBiG BGC0000170). Top, the reference cluster drawn to scale from the *A. niger* locus, with genes colored by function. Dashed lines link each gene to its column below. Left, genome-scale species tree (topology from Figure 2) with the cluster state of each genome: complete ten-gene cluster (green circle), partial locus retaining *yanC*, *yanD* and *yanR* or a subset (orange circle), or no cluster genes at the syntenic locus (red cross). Right, status of each of the ten genes in each genome, based on tBLASTn searches of the *A. niger* ATCC 1015 reference proteins (UniProt G3Y415–G3Y424) against the raw genome sequences and BLASTP searches against the predicted proteomes. Green cells, homolog located at the cluster locus with ≥60% amino acid identity; purple cells with dotted borders, homolog with ≥60% identity located elsewhere in the genome; hatched cells, not detected (best genome-wide hit <60% identity). Values give the higher of the genome-level (tBLASTn) and protein-level (BLASTP) identities to the reference protein (%). (**B**) Chemical structure of yanuthone D (C_28_H_38_O_8_; PubChem CID 139586902).

antiSMASH KnownClusterBlast matched one S23 region to the yanuthone D cluster (MIBiG BGC0000170) at 90% similarity. This cluster was first characterized in *A. niger* and encodes the meroterpenoid yanuthone D, which has reported antifungal activity against *Candida albicans* [12]. The MIBiG entry for this cluster is annotated as of questionable quality and unknown completeness. We therefore defined the cluster according to its primary characterization by Holm et al. [12], comprising ten genes (*yanA–yanI* and the regulatory gene *yanR*), although its exact boundaries remain uncertain. tBLASTn searches of the ten reference proteins (*A. niger* ATCC 1015) against the S23 genome sequence showed that all ten genes are present and co-located within a ~24-kb region of contig NODE_16 (Table S8). In the S23 annotation (g3996.t1–g4004.t1), *yanI* corresponds to g3999.t1 (80% amino-acid identity), and *yanB* and *yanC* are encoded within a single AUGUSTUS model, g4003.t1 (779 amino acids; residues 1–290 and 293–779, respectively). The S23 proteins were 80–97% identical to the reference proteins (Figure 6A). Thus, S23 carries the complete ten-gene cluster defined by Holm et al. [12]. In the absence of metabolite data, these findings indicate the genomic potential of S23 for yanuthone D biosynthesis, although its actual production remains to be confirmed experimentally.

We next examined whether the complete yanuthone D cluster is conserved across the clade or restricted to isolate S23. To avoid errors from ab initio and public gene models, which can split or merge adjacent genes, we searched the ten reference proteins against the raw genome sequence of each panel genome using tBLASTn (E < 1 × 10^−5^). A gene was considered present in the cluster only if its best tBLASTn hit was located in the same genomic region as the other cluster genes. Hits located elsewhere in the genome were not counted as cluster genes. Additional searches using the S23 gene models as queries, including the genome-wide search for the core polyketide synthase *YanA*, are provided in Supplementary Data S9–S13. Only four of the 14 genomes, S23, *A. niger*, *A. welwitschiae* and *A. brasiliensis*, carried the complete ten-gene cluster with conserved gene order (Figure 6). In the other five *A. tubingensis* strains, *A. neoniger* and *A. piperis*, the locus between the conserved flanking genes g3995 and g4005 spanned only ~11–12 kb, which is roughly half of its length in S23 (~24 kb). In *A. luchuensis*, the two flanking genes located on different contigs, so the locus length could not be determined. These seven genomes and *A. luchuensis* retained three cluster genes, *yanC*, *yanD*, and *yanR* (71–93% identity to the reference), whereas the other seven genes, including the core polyketide synthase gene *yanA*, were absent. A genome-wide tBLASTn search with the S23 YanA sequence found only distant paralogs in these eight genomes and in *A. eucalypticola* (27.9–30.4% identity over 60–82% of the query; Table S9; Supplementary Data S11 and S12), which is far below the identity expected for orthologs. *A. eucalypticola* lacked all ten genes at the syntenic locus, where the flanking genes are only ~4.4 kb apart, and the outgroup *A. carbonarius* retained only divergent *yanD* and *yanR* homologs at the locus (67% and 64% identity), together with a *yanC* homolog (65%) located elsewhere in the genome.

Thus, among the six *A. tubingensis* genomes analyzed, only S23 carries the complete cluster. Mean identity of the cluster genes to S23 was not correlated with genome-wide patristic distance (Pearson r = 0.161, *p* = 0.60; Spearman ρ = −0.033, *p* = 0.91; n = 13), which is in line with the patchy, non-phylogenetic distributions of secondary metabolite clusters previously reported in section *Nigri* [28].

We next examined the evolutionary history of the cluster. Mapping the cluster distribution onto the genome-scale species tree (Figure 6A) shows that the complete cluster occurs in the *A. niger*–*A. welwitschiae* clade, in *A. brasiliensis* and in S23. However, S23 is nested within a clade of *A. tubingensis* and its sister species in which every other genome carries only the partial locus or none of the cluster genes. Explaining this distribution by loss alone requires at least six independent deletions of the same seven genes within this clade. Alternatively, a single deletion in the ancestor of the clade would instead require S23 to have subsequently re-acquired the complete cluster. Our data provide two observations relevant to these scenarios. First, the conserved flanking genes indicate that the S23 cluster occupies the same syntenic position as the partial loci, and no transposase or integrase domains were detected nearby. Second, the S23 locus is phylogenetically discordant with the rest of the S23 genome. Genome-wide, S23 proteins are a median 99.7% identical to their *A. tubingensis* HFAT15 orthologs, and the genes immediately upstream of the cluster (g3985–g3995) are 96–100% identical. In contrast, the S23 copies of the retained cluster genes *yanD* and *yanR* are only 86–90% and 71–79% identical to their counterparts in the other *A. tubingensis* strains, and identity remains reduced in the first genes downstream of the cluster (87–96%; Table S10). In maximum-likelihood gene trees, the S23 *yanD* and *yanR* sequences fall outside the strongly supported clade formed by the other *A. tubingensis* strains and their sister species (ultrafast bootstrap 99–100%), grouping instead with *A. brasiliensis* (*yanD*, 89%) or with *A. niger*–*A. welwitschiae* (*yanR*, 99%), whereas an upstream flanking gene places S23 within *A. tubingensis* as expected (Figure S3; Supplementary Data S14). These patterns are not consistent with simple vertical retention of an ancestral *A. tubingensis* allele. They are compatible with acquisition of the S23 locus by introgression or horizontal transfer from a section *Nigri* lineage not represented in our panel, followed by recombination into the syntenic position, or with long-term maintenance of divergent alleles. Our data cannot distinguish between these scenarios, and horizontal transfer cannot be excluded [36,37]. Population-level sampling of *A. tubingensis* would be needed to resolve the origin of this locus.

#### 2.5.2. Burnettiene A Biosynthetic Gene Cluster: Comparative and Evolutionary Analysis

The gene cluster for burnettiene A, a polyene-decalin polyketide with reported cytotoxic and antifungal activity [13,35], was identified with complete 100% similarity for isolate S23 assembly. All 8 genes of the reference cluster (MIBiG BGC0002139) matched at high identity with almost complete coverage (96.5–102.5%). This reference cluster was originally reported from *A. burnettii*, which belongs to section *Flavi* (subgenus *Circumdati*), taxonomically distant from *A. tubingensis*.

**Figure 7:**
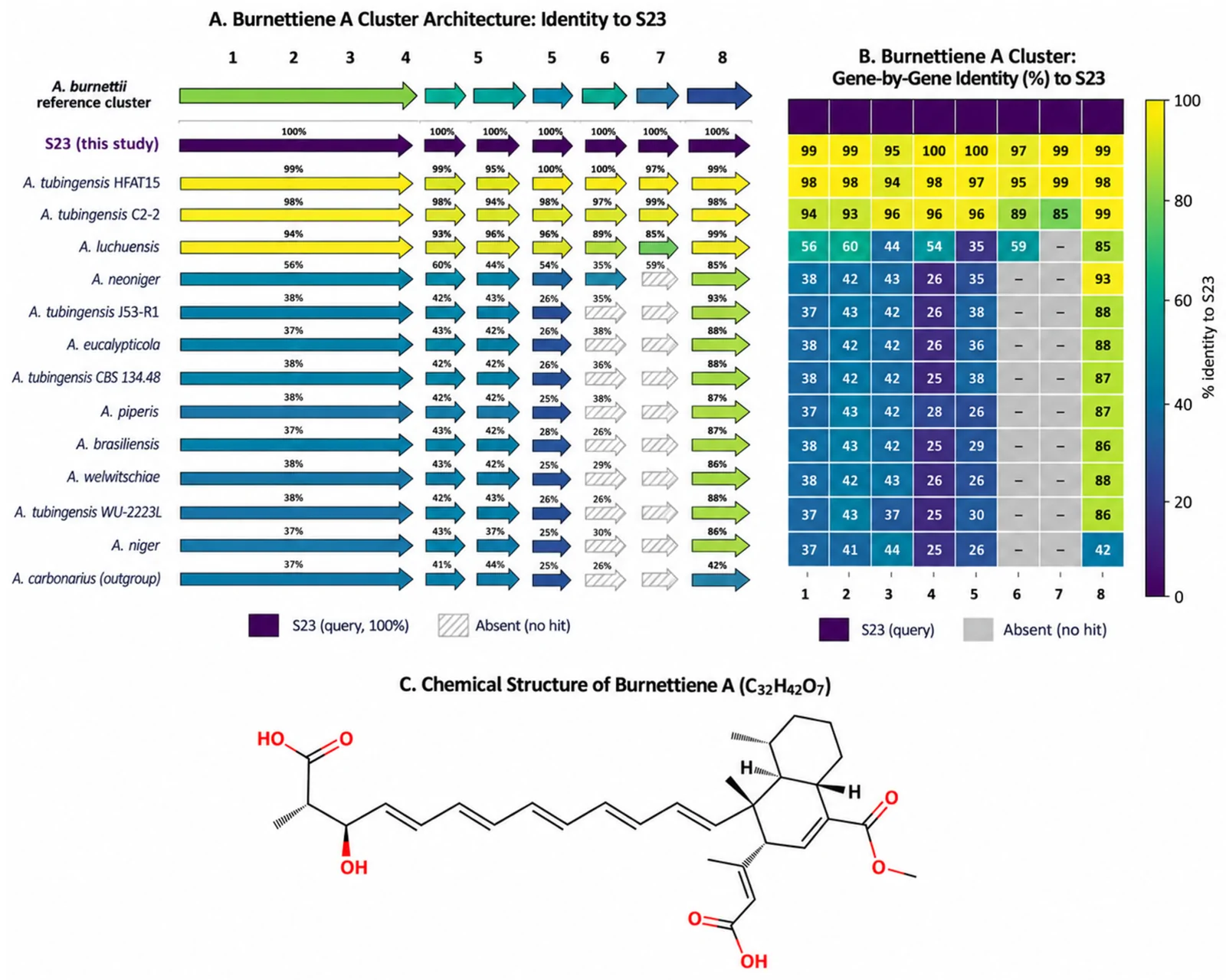
Burnettiene A biosynthetic gene cluster comparative and evolutionary analysis. (**A**) Gene architecture across all 14 genomes compared to the *A. burnettii* reference cluster (BGC0002139); arrow widths are proportional to coding sequence length. Arrows are color-scaled by percent identity to isolate S23 (query, dark purple). (**B**) Corresponding gene-by-gene identity heatmap; genomes are ordered by mean identity (completeness). Gene numbers 1–8 correspond to isolate S23’s own AUGUSTUS gene predictions (g8345.t1–g8352.t1). Gene 1 (g8345) is the core polyketide synthase; genes 2–8 (g8346–g8352) are annotated as “hypothetical protein” in the MIBiG reference cluster. (**C**) Chemical structure of burnettiene A (C32H42O7), confirmed against the original characterization report from Roux et al. [13].

The antiSMASH scan followed by tBLASTn verification across the 13 reference genomes identified a patchy, uneven presence of the Burnettiene A coding cluster across the clade (Supplementary Data S15–S17). The complete, high-identity cluster was confirmed only in isolate S23, two *A. tubingensis* strains (C2-2, HFAT15), and the sister species *A. luchuensis*. The remaining three reference *A. tubingensis* strains (CBS 134.48, WU-2223L, JS3-R1) showed weak and consistently low identity, i.e., 35–46%, indicating that these strains carry only non-orthologous (paralogous) matches. This represents a fine-grained intraspecific split within the *A. tubingensis* strains, and a complete Burnettiene A coding cluster is present in some conspecific strains (Figure 7).

A dedicated maximum-likelihood phylogenetic analysis was performed to test whether the observed distribution pattern of the Burnettiene A gene cluster among species originated via differential loss from an ancestrally present cluster, or horizontal gene transfer between specific lineages. The tree was reconstructed based on core large polyketide synthase gene (g8345) sequences from four genomes that carry the complete cluster (Supplementary Data S18–S20). The resulting tree topology clearly supported a sister relationship between isolate S23 and *A. tubingensis* HFAT15 (100% bootstrap), congruent with the close relationship between these two as detected in the earlier whole-genome phylogeny. However, the relationship of *A. tubingensis* C2-2 and *A. luchuensis* to this pair could not be resolved due to a basal trifurcation, and therefore the deeper topology could not be further compared to the species tree at this node (Figure S4). The S23/HFAT15 grouping, which mirrors the species tree, is consistent with vertical inheritance at this node, although a single gene tree with an unresolved basal trifurcation cannot exclude other scenarios. Fungal secondary metabolite gene clusters are reported to have a tendency toward relaxed selection and repeated independent loss [38,39]. Based on this literature and the current analysis, the burnettiene A cluster may have been present in a common ancestor of this group and subsequently lost several times across the panel, while retained intact in isolate S23, two reference strains, and one sister species. No positive evidence for horizontal transfer was found, though a sufficiently ancient event cannot be formally excluded.

#### 2.5.3. Kojic Acid Biosynthetic Gene Cluster: Comparative and Evolutionary Analysis

One region of the isolate S23 assembly showed partial similarity (66%) to the reference kojic acid biosynthetic gene cluster (MIBiG BGC0002195.2). The reference cluster comprises three genes: the *kojR* Zn(II)_2_Cys_6_ transcription factor, the *kojT* MFS transporter, and the FAD-dependent oxidoreductase *kojA*, originally identified in *A. oryzae* [33]. The isolate S23 showed homology only to *kojR* and *kojT* genes at 61% and 77% identity, respectively, but no homology was detected to the core biosynthetic kojA enzyme. A similar partial kojic acid BGC signature has been reported for *Aspergillus niger* [32,40].

**Figure 8:**
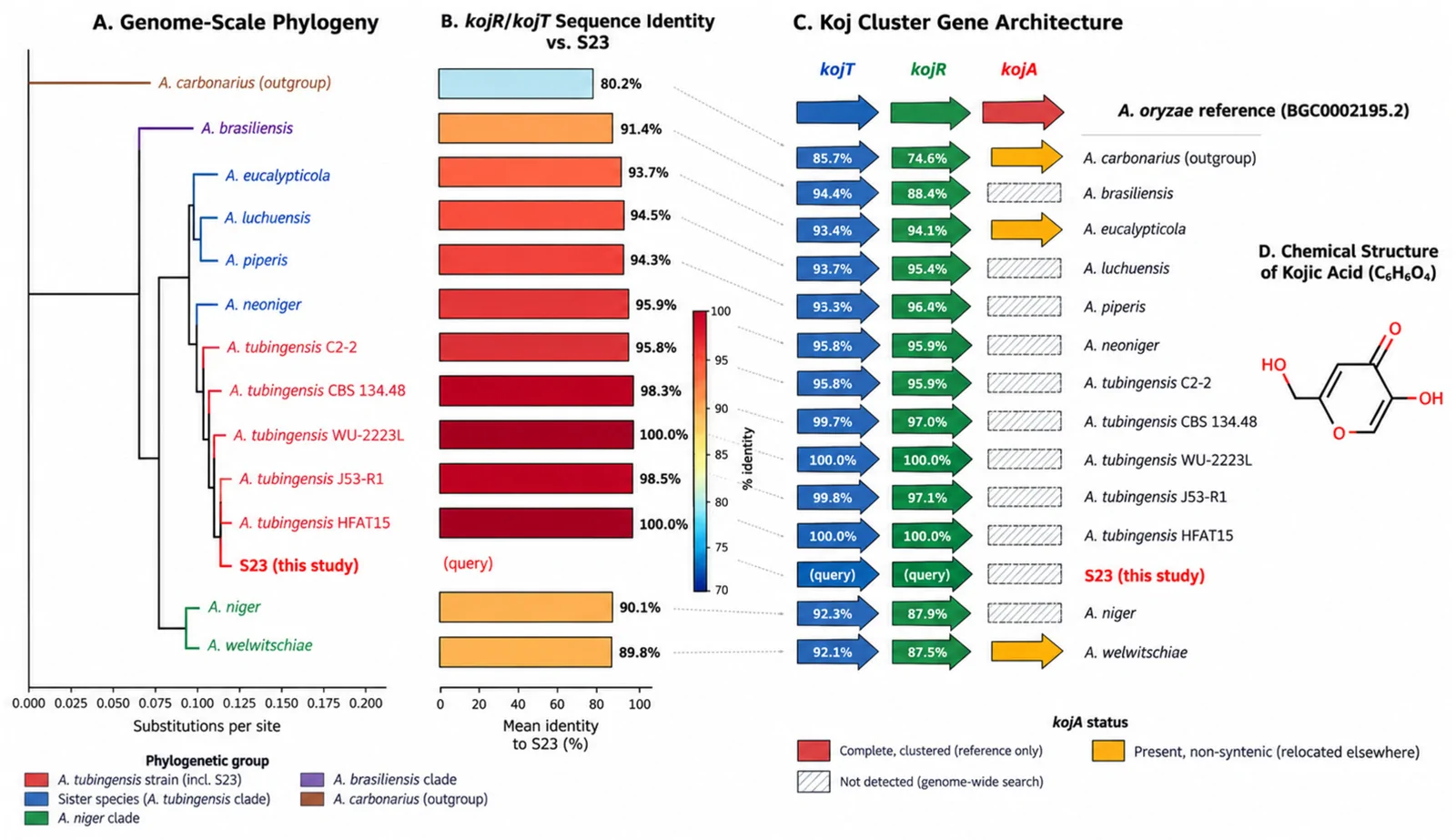
Kojic acid biosynthetic gene cluster comparative and evolutionary analysis. (**A**) Genome-scale phylogeny (OrthoFinder). (**B**) Mean sequence identity of each reference genome’s *kojR*-like and *kojT*-like genes relative to S23 (Pearson r = −0.945, *p* = 0.000001; Spearman ρ = −0.966, *p* < 0.0001; n = 13). (**C**) Gene architecture across all 14 genomes compared to the characterized *A. oryzae* reference cluster. kojA status is shown in three states: complete and clustered (reference only, red); present as a genuine ortholog but not co-located with *kojR*/*kojT* (amber, three genomes: *A. carbonarius*, ~72% identity; *A. eucalypticola*, ~71% identity; *A. welwitschiae*, ~70% identity); and not detected by comprehensive genome-wide search (dashed gray box). (**D**) Chemical structure of kojic acid (C_6_H_6_O_4_; RDKit-rendered, §4.9; PubChem CID 3840, ChEBI:43572).

A comparative investigation across all 14 panel genomes confirmed this partial cluster, with an essentially identical signature, including the outgroup, *A. carbonarius* (Figure 8A,B; Supplementary Data S21). In panel genomes, the sequence identity of the *kojR/kojT* partial cluster to isolate S23 is congruent closely with genome-scale phylogenetic distance (Pearson r = −0.945, *p* = 0.000001; Spearman ρ = −0.966, *p* < 0.0001; n = 13 references). This is consistent with vertical inheritance of this partial cluster across the panel.

The consistent retention of *kojR* and *kojT* across the entire panel of genomes raised a question of whether *kojA* might likewise be retained but relocated elsewhere in the genome, rather than genuinely lost. To test this, the kojA reference protein (UniProt Q2U5I0) was searched against the predicted proteomes of all 14 panel genomes using BLASTP at stringent (E < 1 × 10^−5^) and permissive (E < 1.0) thresholds. The locus was also searched against the raw genome sequences by tBLASTn (E < 1 × 10^−5^) to exclude annotation artifacts. Neither search detected *kojA* in isolate S23 or in ten of the thirteen panel genomes. At the permissive threshold, the matches recovered in these genomes were short (7–25% query coverage) and showed low identity to unrelated FAD-monooxygenase family enzymes, rather than genuine *kojA* orthology (Supplementary Data S22).

In contrast, three genomes, *A. eucalypticola*, *A. welwitschiae*, and the outgroup *A. carbonarius* showed strong, unambiguous hits with 69–72% identity across 98% of the query length, with E-values of 0 to 1.85 × 10^−177^. A neighbor-joining tree constructed from these three candidates, the *kojA* reference (Supplementary Data S23), and five confirmed paralogs of known alternative function (salicylate hydroxylase, squalene epoxidase, steroid and flavin monooxygenase; Supplementary Data S24) placed all three candidates in a single, well-separated clade with the true *kojA* reference (~70% pairwise identity), clearly distinct from the paralog clade (22–46% identity to *kojA*) (Figure S5). This confirms that these three panel species carry true *kojA* orthologs rather than more distant family members.

To test whether *kojA* remains clustered with *kojR*/*kojT*, the genomic positions of the three *kojA* orthologs were compared with those of *kojR*/*kojT* in the same genomes. In all three, *kojA* lies on a different contig from *kojR*/*kojT* (Figure 8C). The *kojR/kojT* genes lie at least 276 kb from the nearest contig end, and *kojA* lies 18–398 kb from the nearest end of its contig. Thus, even if the contigs were joined at their nearest ends, *kojA* would still be at least 496 kb from *kojR/kojT* in *A. eucalypticola*, 371 kb in *A. welwitschiae*, and 850 kb in *A. carbonarius*. Thus, *kojA* is not co-located with *kojR*/*kojT* in these assemblies. As these are draft assemblies without chromosome-level scaffolding or synteny information around *kojA*, we describe *kojA* as unlinked from *kojR*/*kojT* rather than as relocated. The origin of this separation remains unresolved.

#### 2.5.4. Comparative Analysis of the Nidulanin A BGC

antiSMASH identified a region in the S23 assembly that matched the nidulanin A reference cluster (MIBiG BGC0001699) at 75% similarity. This region required closer investigation because nidulanin A has an unusual biosynthetic basis. Andersen et al. [34] showed that nidulanin A is produced by two physically unlinked genes on different chromosomes: the NRPS gene (AN1242/nlsA, chromosome VIII) and a prenyltransferase gene (AN11080/nptA, chromosome V) whose product N-prenylates the NRPS product. The MIBiG reference cluster covers only the local region around the NRPS, comprising an ABC transporter, the NRPS itself, and an MFS transporter, and these three genes matched the three genes of the S23 cluster. Because the prenyltransferase is located on a separate chromosome, a region-based search cannot determine whether it is present in S23 or the other genomes. We therefore searched the S23 proteome for homologs of the *A. nidulans* FGSC A4 prenyltransferase NptA (gene AN11080; UniProt C8VEJ5) by BLASTP. The top hit, g7212.t1, was a full-length match (99% query coverage, 32.9% identity, E-value = 8.99 × 10^−78^), and a weaker second hit, g10463.t1, was also detected (25.9% identity, 73% coverage; Supplementary Data S25 and S26). Because NptA belongs to a large and functionally diverse prenyltransferase family with many substrate-specific paralogs in *Aspergillus*, orthology cannot be inferred from sequence identity alone. Both candidates were therefore included in a phylogenetic tree with the NptA reference and two functionally characterized paralogs from the same family (FgaPT2/DmaW, the tryptophan prenyltransferase of *A. fumigatus*, and TdiB, the terrequinone A prenyltransferase of *A. nidulans*). S23 g7212.t1 clustered with NptA (41.2% pairwise identity), more closely than NptA did with either FgaPT2 or TdiB (Figure S6), supporting its identification as the NptA ortholog. The weaker candidate, g10463.t1, fell outside all reference sequences and is therefore unlikely to be an ortholog.

**Figure 9:**
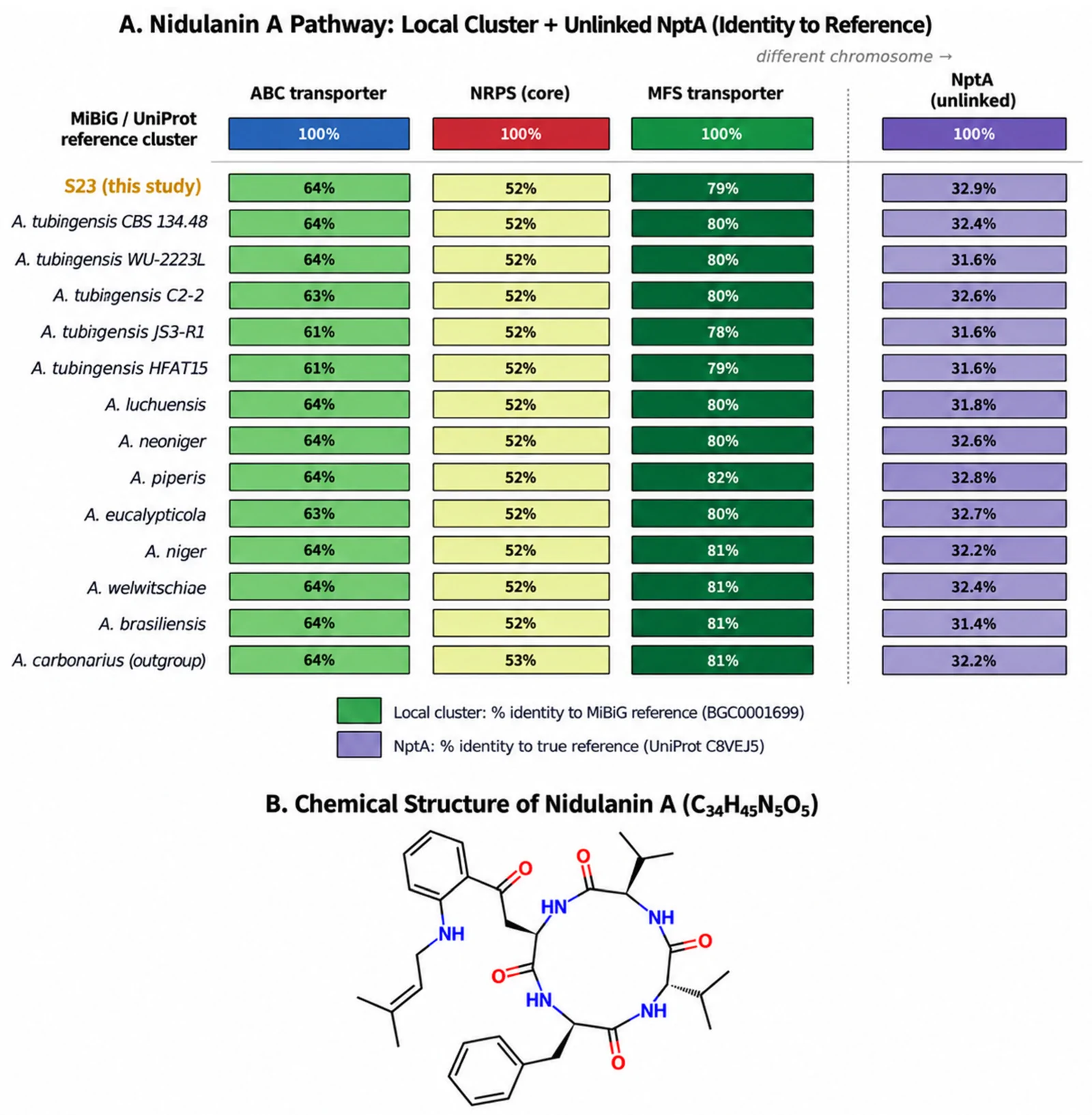
Nidulanin A pathway comparative and evolutionary analysis. (**A**) Percent identity to the reference, shown separately for the local 3-gene cluster (ABC transporter, NRPS core, MFS transporter; MIBiG BGC0001699) and the physically unlinked NptA gene (UniProt C8VEJ5). Reference row (100%, distinct colors) represents the source database entries themselves, not a genome comparison. “different chromosome →” means NptA is located on different chromosome. (**B**) Chemical structure of nidulanin A (C34H45N5O5), a cyclic tetrapeptide (L-Phe–L-Kyn–L-Val–D-Val) bearing an isoprenyl group N-linked to the kynurenine side-chain amine. The structure shown was reconstructed directly from the explicit sequence and modification-site description given in the original characterization paper [34], and the structure was rendered using RDKit.

Both components of the nidulanin A biosynthesis were then examined across the 14-genome panel, with NptA searched against the genome sequences of the other 13 genomes by tBLASTn (Supplementary Data S27). The local NRPS cluster and the unlinked prenyltransferase showed similar identities in all genomes, including the outgroup *A. carbonarius*, with no relationship to phylogenetic distance from S23 (Figure 9; Table S11; Supplementary Data S25–S30). The presence of both pathway components in every panel genome, at uniformly high identity, indicates that the pathway is conserved across the panel, consistent with previous reports for *Aspergillus* [34]. Because no codon-based (dN/dS) analysis was performed, this conservation alone cannot be attributed to a specific type or strength of selection on these genes.

#### 2.5.5. TAN-1612 Biosynthetic Gene Cluster: Comparative and Evolutionary Analysis

**Figure 10:**
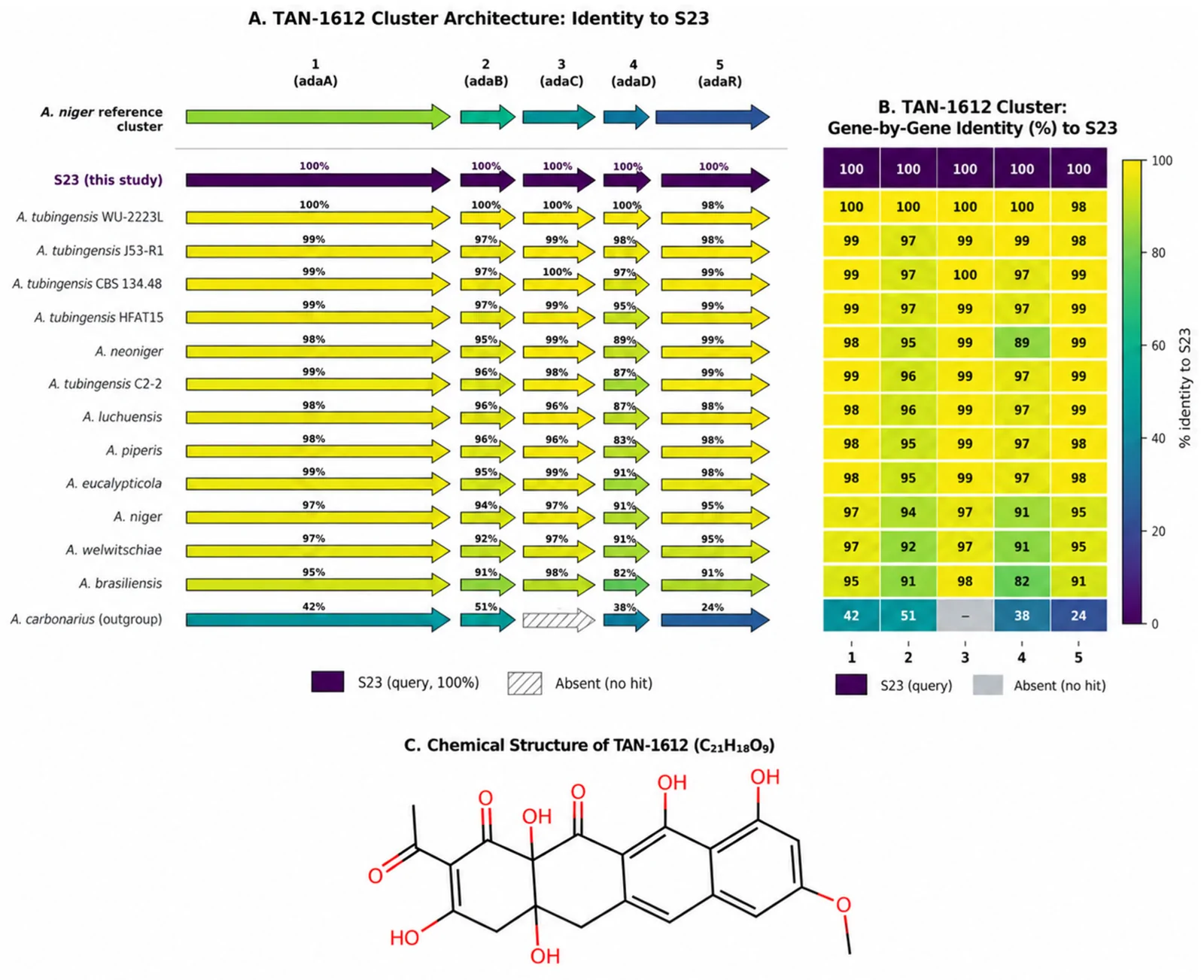
TAN-1612 biosynthetic gene cluster comparative and evolutionary analysis. (**A**) Gene architecture across all 14 genomes compared to the *A. niger* reference cluster (top row); arrow widths are proportional to coding sequence length. Numbered columns 1–5 correspond to isolate S23’s own AUGUSTUS gene predictions g1713.t1–g1717.t1, matching the characterized adaA, adaB, adaC, adaD, and adaR genes, respectively (MIBiG BGC0000156). (**B**) Corresponding gene-by-gene identity heatmap, genomes ordered by mean identity. (**C**) Chemical structure of TAN-1612 (C21H18O9; RDKit-rendered).

TAN-1612 BGC region match was high-confidence for isolate S23, and all five genes, including core biosynthetic genes (adaA–adaD) and the regulator (adaR) gene, showed high identity (91–97%) and nearly complete coverage (90.7–100%) to the reference cluster (MIBiG BGC0000156). TAN-1612 (BMS-192548) was originally isolated from *Aspergillus niger* WB2346 [14]. The gene cluster network analysis across the genus reported this cluster as uniformly present throughout both the *A. niger* and *A. tubingensis* clades [41], and a closely homologous cluster (80% similarity) was also identified in a geographically distant *A. tubingensis* strain from a French Mediterranean vineyard [42]. Our results support this observation, as the mean identity across all five TAN-1612 BGC genes was closely congruent with genome-wide phylogenetic distance from S23 (Pearson r = −0.947, *p* = 0.000001; Spearman ρ = −0.949, *p* = 0.000001; n = 13), with nearly uniform 90–100% identity across section Nigri (Figure 10; Supplementary Data S31 and S32). This strong phylogenetic correlation is consistent with vertical inheritance, and no loss of TAN-1612 cluster genes was detected in the panel.

## 3. Discussion

Isolate S23 was identified as *Aspergillus tubingensis* by three congruent lines of evidence. ITS barcoding placed it in section *Nigri*, and both the concatenated (*benA,* CaM, RPB2) tree and the genome-scale phylogeny based on 5395 single-copy orthologs grouped it with *A. tubingensis*, including the ex-type strain, with full support. The isolate is therefore referred to as *A. tubingensis* S23.

*A. tubingensis* S23 carries an extensive and mostly uncharacterized biosynthetic repertoire (95 BGCs, 68.4% without a MIBiG match) and a rich CAZyme complement, both typical of section *Nigri* [4,28]. Direct comparison with close relatives’ genomes showed that most of its BGC families are shared across *A. tubingensis* and its sister species, and that BGC sharing tracks genome-wide phylogenetic distance. Thus, only a limited number of loci differentiate S23 from the reference strains. Among these, the yanuthone D biosynthetic gene cluster represents the most distinctive feature. Only S23 is the *A. tubingensis* genome in the panel with the complete ten-gene cluster, whereas the other five strains and the sister species retain only three of its genes at the same locus. Because the S23 locus is phylogenetically discordant with the rest of its genome, its presence is not explained by simple vertical retention, and acquisition by introgression or horizontal transfer remains possible [36,37]. The burnettiene A cluster showed a second, patchy intraspecific pattern, being complete in S23 and in two of the other five *A. tubingensis* strains, consistent with repeated loss of secondary metabolite clusters [38,39]. In addition, one T1PKS cluster has a core synthase with no ortholog in the panel and is specific to S23. Given the reported antifungal and cytotoxic activities of yanuthone D and burnettiene A [12,13,35], these clusters are priority targets for metabolite profiling to determine whether S23 produces the corresponding pharmaceutically relevant metabolites.

The isolate S23 was recovered from healthy, asymptomatic tissue of *E. isochila*, and its genome is consistent with an endophytic lifestyle. It lacks the ochratoxin A cluster and other complete mycotoxin clusters characteristic of virulent section *Nigri* strains [29]. The plant cell wall degrading enzyme profile of S23 is not enriched in families associated with necrotrophic tissue maceration. Its secretome is dominated by apoplastic effector candidates, with few matches to experimentally confirmed plant-pathogen effectors. The secreted LysM-domain proteins and the GH18 chitinase and GH16 β-1,3-glucanase complement occur in both pathogenic and mutualistic fungi [18,19,26,27] and are therefore candidate, rather than diagnostic, determinants of host and fungal interactions. Expression profiling during colonization, antifungal assays, and isolation of the metabolites encoded by the distinctive BGCs are needed to test these predictions.

## 4. Materials and Methods

### 4.1. Isolation of Endophytic Fungi

The healthy leaves and stem material of *Eremostachys isochila* (*Lamiaceae*) were collected from the Bustonlik district, Tashkent region, Uzbekistan. The specimen was transported to the laboratory and immediately processed for endophytic fungal isolation, as described previously [43]. The Isolate S23 is maintained in the Laboratory of Biotechnology and Bioinformatics, Institute for Advanced Studies, New Uzbekistan University (Tashkent, Uzbekistan), on potato dextrose agar slants at 4 °C and as glycerol stocks at −80 °C.

### 4.2. Genomic DNA Purification and Internal Transcribed Spacer (ITS) Region Sequencing

Isolate S23 was recovered as a fungal endophyte from *E. isochila*. Genomic DNA was extracted using the E.Z.N.A. Tissue DNA Kit (Omega Bio-Tek, Norcross, GA, USA). DNA concentration and purity were assessed using a NanoDrop 2000 spectrophotometer (Thermo Fisher Scientific, Waltham, MA, USA) and a Qubit^®^ 4.0 Fluorometer with the dsDNA HS Assay Kit (Thermo Fisher Scientific). The ITS region was amplified under standard PCR conditions using the ITS5 (forward: 5′-GGAAGTAAAAGTCGTAACAAGG-3′) and ITS4 (reverse: 5′-TCCTCCGCTTATTGATATGC-3′) primers. Amplicons were purified and Sanger-sequenced by Tech Dragon Ltd. (Hong Kong, China).

### 4.3. Whole-Genome Sequencing, Assembly, and Annotation

Sequencing libraries were prepared using the MGI Easy FS DNA Library Preparation Kit (V2.1) and subjected to whole-genome sequencing on an MGI DNBSEQ-T7 platform (MGI Tech, Hong Kong SAR, China) at the Bio-CRF facility, Hong Kong University of Science and Technology (HKUST), generating 2 × 150 bp paired-end reads (mean insert size 274 bp). Raw paired-end reads were quality-trimmed and filtered using fastp [25]. The high-quality reads were assembled using SPAdes v3.15.5 [44,45] in --isolate mode (k-mer sizes 21, 33, 55, 77, 99, 127; coverage cutoff 10). Sequencing produced approximately 20.05 million read pairs (2 × 150 bp; ~6.0 Gb), corresponding to a mean sequencing depth of approximately 169× over the 35.63-Mb assembly. The sequencing reads are available in the NCBI SRA (SRR41331962).

Assembly contiguity metrics were computed with QUAST [46]. Genome completeness was assessed with BUSCO v5.8.0 [47] against the eurotiales_odb10 lineage dataset (n = 4191 BUSCOs) in genome mode. To screen for contamination, the per-contig GC content and coverage (acquired from SPAdes contig headers) were compared across the assembly to identify contigs showing deviation from the genome-wide GC/coverage distribution. The outlier contigs were further analyzed via BLASTn against the NCBI nt database [48] to identify their taxonomic origin. The protein-coding genes were predicted across the draft genome assembly using AUGUSTUS v3.5.0 [49] with *Aspergillus nidulans* as model species, without extrinsic hints (ab initio prediction). Gene models in key BGCs were verified by tBLASTn searches against the genomic sequence (Section 4.8 and Section 4.10).

### 4.4. ITS and Multi-Locus Sequence-Based Species Identification and Phylogenetic Placement

A 394 bp ITS sequence was queried against the NCBI core_nt database using BLASTn (megablast). As ITS do not reliably resolve species within *Aspergillus* section Nigri [7], three additional protein-coding loci were used for species-level identification. The sequences of these protein-coding genes were recovered directly from the S23 genome assembly by *in silico* primer mapping. The reported PCR primer sequences specific for each locus were searched against the assembly using BLASTn in short-sequence mode (word size 7), which is more reliable for short queries. The benA sequence was extracted from the whole-genome assembly using the Ben2f [50] and Bt2b [51] primer set. The standard Bt2a forward primer was replaced with Ben2f, as Bt2a is reported to often mis-amplify the tubC paralog instead of benA [50]. Likewise, the CaM and RPB2 sequences were extracted using the CMD5/CMD6 [52] and fRPB2-5F/fRPB2-7cR [53] primer sets, respectively. A locus was considered to be correctly retrieved when its respective forward and reverse primers mapped to the same contig at a distance matching the expected amplicon size. The validated regions were then extracted and queried against the NCBI core_nt database using BLASTn to check the species identity. Reference sequences for benA, CaM, and RPB2 were obtained from ex-type or reference strains of four *Aspergillus* section *Nigri* species, i.e., *A. tubingensis* (NRRL 4875), *A. niger* (NRRL 326), *A. welwitschiae* (CBS 139.54), and *A. brasiliensis* (CBS 101740). Each locus was aligned with MAFFT v7.505 [54], using “--auto --adjustdirection” because the benA and CaM sequences extracted from Isolate S23 genome were reverse-complement to the references. All aligned loci (benA, 602 bp; CaM, 746 bp; RPB2, 1220 bp) were concatenated into a 2568 bp supermatrix. *A. welwitschiae*’s RPB2 1014 bp CDS partial fragment (GenBank MN969100) aligned with no internal gaps. A partitioned maximum-likelihood phylogeny was inferred using IQ-TREE v2.0.7 [55] with ModelFinder (-m MFP) for per-partition model selection and 1000 ultrafast bootstrap replicates.

### 4.5. Reference Genome Panel Selection and Genome-Scale Phylogenomics

A panel of 13 reference genomes was shortlisted for genome-scale phylogenomic inference of isolate S23. Panel selection was based on two criteria: taxa that returned as close BLAST hits but were not included in the former 3-locus-based phylogenetic analysis, and taxa relevant to the ongoing section Nigri species-boundary debate in the literature [9,10]. The outgroup was likewise selected on precedent from the section *Nigri* phylogenetic literature [9]. The final panel comprised five *Aspergillus tubingensis* strains (CBS 134.48, WU-2223L, C2-2, JS3-R1, and HFAT15), *Aspergillus neoniger*, *Aspergillus luchuensis*, *Aspergillus piperis*, *Aspergillus eucalypticola*, *Aspergillus niger*, *Aspergillus welwitschiae*, *Aspergillus brasiliensis,* and *Aspergillus carbonarius* (as outgroup). Reference proteomes were retrieved from NCBI RefSeq and GenBank annotations. Three *A. tubingensis* strains lacking official annotation were annotated locally using AUGUSTUS. Orthologous groups and a genome-scale species tree were inferred using OrthoFinder v3.1.5 [56] from the predicted proteomes of isolate S23 and the reference genomes panel. OrthoFinder was used based on default parameters, which employ DIAMOND for the initial all-vs-all sequence comparison, MCL for orthogroup clustering, FAMSA for within-orthogroup sequence alignment, FastTree for individual gene tree construction [57], and STRIDE for rooting the final tree [58].

### 4.6. CAZyme, Secretome, Effector and Virulence-Factor Annotation

Carbohydrate-active enzymes (CAZymes) were annotated using the predicted proteome with run_dbcan v5.2.9 [59] by combining DIAMOND [60] search against the CAZy database [61] (E-value < 1 × 10^−102^) with HMMER [62] search against the dbCAN HMM and dbCAN-sub databases (E-value < 1 × 10^−15^, coverage > 0.35) for subfamily and substrate-level classification. CAZymes supported by at least two of these three searches were considered high-confidence calls, and the same procedure was applied to all 13 reference proteomes.

Signal peptides were predicted with SignalP 6.0 [22] (fast mode, organism: Eukarya), and the predicted secreted proteins were screened for effector-like properties with EffectorP-fungi 3.0 [23] (Supplementary Data S2). The secretome was searched with BLASTP (E < 1 × 10^−10^) against PHI-base [21] and DFVF [24]. PHI-base matches were cross-referenced with the PHI-base metadata to retrieve the host, disease, and mutant phenotype of each matched gene (Supplementary Data S3). LysM-domain proteins were identified from Pfam domain annotations (PF01476) obtained with eggNOG-mapper v2 [63] and from dbCAN CBM50 assignments.

### 4.7. Secondary Metabolite Biosynthetic Gene Cluster (BGC) Detection

Secondary metabolite biosynthetic gene clusters were identified using antiSMASH v8.0.4 [15] in fungal mode, with KnownClusterBlast, ClusterBlast, MIBiG cluster comparison, SubClusterBlast, and Pfam-based annotation modules. Gene models for all genomes, including isolate S23, were pre-computed and supplied directly to antiSMASH via “--genefinding-gff3”, instead of relying on antiSMASH’s own internal gene-finding tool. Isolate S23 used its AUGUSTUS-derived annotation.

### 4.8. Gene Cluster Family (GCF) Network Analysis

BiG-SCAPE v2.0.3 [64] was used to group BGCs into gene cluster families based on Pfam domain content, following a distance cutoff of 0.3 and antiSMASH class-based binning. Isolate S23’s BGCs were compared against the MIBiG v3.1 [16] reference database of experimentally characterized clusters, as well as against the 13 reference genomes described in Section 4.5. Gene cluster families found only in S23 were verified by searching each encoded protein against the raw genome sequence of every panel genome (tBLASTn, E < 1 × 10^−10^) and the core biosynthetic enzymes against each proteome (BLASTP, E < 1 × 10^−10^). A core enzyme with ≥70% identity was taken as evidence of an ortholog.

### 4.9. Chemical Structure Verification

Chemical structures shown were independently sourced from PubChem [65], ChEBI [66], DrugBank [67], and KEGG [68] where available. The chemical formulas were verified against source data and rendered as 2D skeletal diagrams using RDKit v2026.3.5 [69].

### 4.10. Genome-Level Verification of BGC Gene Content and Gene Trees

S23 and three reference genomes were annotated ab initio; the gene content of the yanuthone D and kojic acid loci was verified independently of annotation. Reference proteins for the yanuthone D cluster (*A. niger* ATCC 1015 YanA–YanI and YanR; UniProt G3Y415–G3Y424) and the kojic acid cluster (*A. oryzae* RIB40 KojA, KojR and KojT; UniProt Q2U5I0, Q2U5H9 and Q2U5H8) were searched against the raw genome sequence of each panel genome with tBLASTn (BLAST+ [48]; E < 1 × 10^−5^). HSPs within 15 kb of the best hit were merged to calculate locus-level identity and query coverage, and a gene was scored as present at a cluster locus when its best hit was co-located with the other cluster genes. Protein-level identities were obtained by BLASTP against each proteome. For yanD, yanR, and the upstream flanking gene g3995, the S23 proteins were searched against each proteome with BLASTP (E < 1 × 10^−20^), and the best hits were aligned with MAFFT v7 [54]. Maximum-likelihood trees were inferred with IQ-TREE v3.1.3 [55] using ModelFinder and 1000 ultrafast bootstrap replicates, with *A. carbonarius* as the outgroup. *A. eucalypticola* was excluded from this analysis. It lacks all yanuthone D cluster genes, and its best hits for yanD and yanR were distant paralogs (36% and 22% identity). Genome-wide background identity between S23 and *A. tubingensis* HFAT15 was estimated as the median identity of the BLASTP best hits (≥90% query coverage) for a systematic subsample of the S23 proteome (i.e., every 30th predicted protein; n = 358).

## 5. Conclusions

This study presents whole-genome sequencing and functional annotation of isolate S23, an endophytic fungus recovered from healthy *Eremostachys isochila* tissue. The ITS-based section-level placement, concatenated multi-locus phylogeny, and genome-scale ortholog-based phylogenomics confirm the isolate’s identity as *Aspergillus tubingensis*. This identification is further supported by CAZyme and BGC contents broadly tracking phylogeny across the panel reference genomes.

Genome mining revealed a substantial secondary-metabolite repertoire comprising 95 predicted BGCs, of which 68.4% lacked matches to characterized MIBiG clusters, highlighting considerable uncharacterized biosynthetic diversity. Comparative analysis identified three loci that distinguish S23 within the panel. S23 is the only *A. tubingensis* genome with the complete yanuthone D cluster, and this locus is phylogenetically discordant with the rest of its genome. S23 also carries a T1PKS cluster whose core polyketide synthase has no ortholog in any other panel genome. The burnettiene A cluster, by contrast, is shared with only some of its closest relatives, revealing patchy intraspecific variation. In contrast, the nidulanin A and TAN-1612 clusters were conserved across the panel, whereas *kojA*, the core gene of kojic acid biosynthesis, was missing from every *A. tubingensis* genome examined, including S23. Overall, these findings demonstrate that closely related *A. tubingensis* strains can differ substantially in selected secondary-metabolite loci despite strong genome-wide similarity. The distinctive BGCs of S23 provide targets for metabolomic and functional validation.

## Figures and Tables

**Table 1:** Biosynthetic gene clusters in isolate S23 with similarity to characterized compounds of reported pharmaceutical, cosmetic, or antimicrobial potential.

Compound	MIBiG Similarity	Chemical Class	Documented/Reported Bioactivity of the Reference Compound [Reference]
Kojic acid	66%	Pyranone	Skin-whitening (cosmetic), antioxidant and antimicrobial [32,33]
Yanuthone D	90%	Meroterpenoid	Antifungal (*Candida albicans*) [12]
Clavaric acid	100%	Triterpenoid	Antitumor [30]
TAN-1612	100%	Anthraquinone-type polyketide	Neuropeptide Y receptor binding inhibitor (BMS-192548) [14]
Nidulanin A	75%	Polyketide–nonribosomal peptide hybrid	Not characterized [34]
Burnettiene A	100%	Polyketide	Cytotoxic (mouse myeloma NS-1 cells) [13]; antifungal against plant-pathogenic fungi [35]

MIBiG similarity is the antiSMASH KnownClusterBlast score (percentage of genes in the MIBiG reference cluster with significant hits in the S23 cluster). Bioactivities refer to the characterized reference compounds; production of these compounds by S23 has not been tested.

## Data Availability

The whole-genome sequencing project for *Aspergillus tubingensis* S23 has been registered at NCBI under BioProject PRJNA1532414 and BioSample SAMN63274691. The genome assembly has been submitted to GenBank and is scheduled for public release on 1 January 2027 or upon publication, whichever occurs first. The sequencing reads have been deposited in the NCBI Sequence Read Archive under BioProject PRJNA1515412 (BioSample SAMN62582722; run SRR41331962). Other data supporting the findings of this study are provided in the Supplementary Materials.

## Supplementary Materials

The following supporting information can be downloaded at: https://www.mdpi.com/article/10.3390/molecules31213939/s1.

## Abbreviations

The following abbreviations are used in this manuscript:

BGC	Biosynthetic Gene Cluster
BUSCO	Benchmarking Universal Single-Copy Orthologs
CAZyme	Carbohydrate-Active Enzyme
GCF	Gene Cluster Family
ITS	Internal Transcribed Spacer
MIBiG	Minimum Information about a Biosynthetic Gene Cluster
NRPS	Nonribosomal Peptide Synthetase
PKS	Polyketide Synthase
T1PKS	Type I Polyketide Synthase
