# Supplementary material for: Whole-Genome Sequencing Identifies a New Aspergillus tubingensis Strain from Eremostachys isochila with Intraspecific Variation in Pharmaceutically Relevant Biosynthetic Gene Clusters
Source: Molecules. 2026 Nov 1;31(21):3939. doi: 10.3390/molecules31213939 (PMC13765160; doi:10.3390/molecules31213939)
Supplement: Supplementary file 1 [file molecules-31-03939-s001.zip › molecules-4613349-supplementary.pdf]

Supplementary Materials

Whole-Genome Sequencing Identifies a New *Aspergillus tubingensis* Strain from *Eremostachys isochila* with Intraspecific Variation in Pharmaceutically Relevant Biosynthetic Gene Clusters

Supplementary Tables:

Table S1: BLASTn results for isolate S23's partial ITS sequence (394 bp) against the NCBI core_nt database, showing the top hit and representative hits for each tied second-tier species within *Aspergillus section* Nigri.

Species	Identity	Query Coverage	Accession Length (bp)	GenBank Accession
Aspergillus niger	99.24%	100%	610	OR135784.1
Aspergillus welwitschiae	98.98%	100%	568	PX116846.1
Aspergillus tubingensis	98.98%	100%	572	OR392779.1
Aspergillus foetidus	98.98%	100%	599	OR726136.1
Aspergillus awamori	98.98%	100%	1172	LC573568.1
Aspergillus phoenicis	98.98%	100%	556	ON365687.1
Aspergillus costaricensis	98.98%	100%	595	MN198160.1
Aspergillus piperis	98.98%	100%	574	MT529179.1

Table S2: BLASTn results for isolate S23's benA sequence against the NCBI core_nt database, showing the top hit and comparison hits against *A. niger*.

Description	Species	Identity	Query Coverage	Accession
Beta-tubulin, partial cds (NBRC:4050)	*Aspergillus tubingensis*	100.00%	99%	LC573671.1
Beta-tubulin, partial cds (NBRC:31125)	*Aspergillus tubingensis*	99.29%	99%	LC573675.1
Beta-tubulin, partial cds (NBRC:4407)	*Aspergillus tubingensis*	99.29%	99%	LC573673.1
Beta-tubulin gene, complete cds (isolate ASP-01)	*Aspergillus niger*	94.21%	100%	OM937136.1
Beta-tubulin gene, complete cds (culture CBS:513.88)	*Aspergillus niger*	94.21%	100%	MG701893.1
Genomic contig An08c0100	*Aspergillus niger*	94.21%	100%	AM270165.1

Table S3: BLASTn results for isolate S23's CaM sequence against the NCBI core_nt database, showing dedicated calmodulin-gene hits across *Aspergillus section* Nigri species.

Description	Species	Identity	Query Coverage	Accession
Genomic contig An04c0200 (non-dedicated)	*Aspergillus niger*	92.79%	100%	AM270081.1
Calmodulin gene, partial cds (NBRC:7523)	*Aspergillus tubingensis*	100.00%	74%	LC573733.1
Calmodulin (CaM) gene, partial cds (isolate PW3979)	*Aspergillus tubingensis*	100.00%	74%	MT415532.1
Calmodulin gene, partial cds (strain B7-1)	*Aspergillus tubingensis*	100.00%	73%	JX024265.1
Calmodulin (caM) gene (voucher IHEM 6184)	*Aspergillus tubingensis*	100.00%	73%	MH644895.1
Calmodulin (caM) gene (voucher IHEM 25949)	*Aspergillus tubingensis*	100.00%	73%	MH644873.1
Calmodulin gene, partial cds (strain HQ)	*Aspergillus niger*	99.86%	72%	PQ285419.1
Calmodulin (cmd) gene (strain MUM03.05)	*Aspergillus phoenicis*	99.45%	72%	JF838353.1
CaM gene for calmodulin (CBS 115656)	*Aspergillus neoniger*	98.78%	74%	LC570773.1
Calmodulin gene (isolate NRRL 62634)	*Aspergillus neoniger*	98.77%	73%	KC796377.1
Calmodulin gene for calmodulin (NBRC:4338)	*Aspergillus foetidus*	97.43%	74%	LC573702.1

Table S4: BLASTn results for isolate S23's RPB2 sequence against the NCBI core_nt database, showing the top hit and comparison hits against *A. niger*.

Description	Species	Identity	Query Coverage	Accession
RPB2 subunit (AtWU_03698), partial mRNA	*Aspergillus tubingensis*	99.94%	100%	XM_035498810.1
RPB2 (RPB2), partial mRNA	*Aspergillus luchuensis*	98.50%	100%	XM_041682128.1
RPB2 core subunit (BO85DRAFT_441868)	*Aspergillus piperis*	98.50%	100%	XM_025658818.1
Uncharacterized protein (An12g00720), partial mRNA	*Aspergillus niger*	94.88%	100%	XM_059750647.1
RPB2 core subunit, CBS 101883 (BO96DRAFT_123568)	*Aspergillus niger*	94.88%	100%	XM_025592545.1
Genomic contig An12c0030	*Aspergillus niger*	94.88%	100%	AM270260.1

Table S5: Isolate S23 secreted proteins matching PHI-base entries with a confirmed effector phenotype (≥50% identity), with EffectorP-fungi 3.0 prediction.

**S23 gene**	**Matched pathogen**	**Host**	**Disease**	**PHI-base UniProt**	**Identity (%)**	**EffectorP-fungi 3.0**
g1201.t1	*Phytophthora sojae*	Glycine max	stem rot (soybean); root rot (soybean)	G4ZHR2	51.2	Yes (apoplastic)
g1443.t1	*Exserohilum turcicum*	Zea mays	northern corn leaf blight	R0K2C5	50.4	Yes (apoplastic/cytoplasmic)
g3226.t1	*Penicillium expansum*	Malus domestica	blue mold	A0A0A2I2M6	60.7	No
g6141.t1	*Penicillium expansum*	Malus domestica	blue mold	A0A0A2I2M6	60.0	No
g9145.t1	*Phytophthora sojae*	Glycine max	stem rot (soybean); root rot (soybean)	G4ZHR2	57.7	No
g9550.t1	*Sporisorium reilianum*	Zea mays	head smut (maize)	E6ZWS5	63.8	Yes (apoplastic)

Secretome predicted with SignalP 6.0 (n = 919; Supplementary Data 2); BLASTP E < 1e-10 against PHI-base. All 434 PHI-base matches are listed in Supplementary Data 3.

Table S6: Secreted LysM-domain proteins of isolate S23 (n = 17 of 41 LysM-domain proteins), with EffectorP-fungi 3.0 prediction and best PHI-base match.

S23 gene	LysM evidence	EffectorP-fungi 3.0	Best PHI-base match (phenotype; identity)
g522.t1	Pfam LysM (PF01476); CBM50 (dbCAN)	Non-effector	*Cercospora beticola* (effector (plant avirulence determinant); 36.0%)
g694.t1	Pfam LysM (PF01476); CBM50 (dbCAN)	Apoplastic effector	*Fulvia fulva* (effector (plant avirulence determinant); 35.3%)
g1403.t1	Pfam LysM (PF01476); CBM50 (dbCAN)	Non-effector	*No hit*
g1600.t1	Pfam LysM (PF01476); CBM50 (dbCAN)	Apoplastic effector	*Penicillium expansum* (effector (plant avirulence determinant); 34.0%)
g1612.t1	Pfam LysM (PF01476); CBM50 (dbCAN)	Apoplastic effector	*Aspergillus fumigatus* (unaffected pathogenicity; 43.9%)
g2172.t1	Pfam LysM (PF01476); CBM50 (dbCAN)	Apoplastic effector	*Aspergillus fumigatus* (unaffected pathogenicity; 54.5%)
g2556.t1	Pfam LysM (PF01476)	Apoplastic effector	*Fulvia fulva* (effector (plant avirulence determinant); 32.2%)
g3633.t1	Pfam LysM (PF01476); CBM50 (dbCAN)	Non-effector	*Cercospora beticola* (effector (plant avirulence determinant); 33.8%)
g3941.t1	CBM50 (dbCAN)	Non-effector	*No hit*
g4012.t1	Pfam LysM (PF01476); CBM50 (dbCAN)	Non-effector	*Fusarium verticillioides* (unaffected pathogenicity; 27.3%)
g4483.t1	CBM50 (dbCAN)	Non-effector	*Cercospora beticola* (effector (plant avirulence determinant); 34.6%)
g4539.t1	Pfam LysM (PF01476); CBM50 (dbCAN)	Apoplastic effector	*Fulvia fulva* (effector (plant avirulence determinant); 37.4%)
g5323.t1	Pfam LysM (PF01476)	Apoplastic effector	*Fulvia fulva* (effector (plant avirulence determinant); 36.2%)
g6048.t1	CBM50 (dbCAN)	Non-effector	*Fusarium graminearum* (unaffected pathogenicity; 62.1%)
g9285.t1	Pfam LysM (PF01476)	Apoplastic effector	*Fulvia fulva* (effector (plant avirulence determinant); 34.8%)
g9503.t1	Pfam LysM (PF01476); CBM50 (dbCAN)	Non-effector	*Beauveria bassiana* (effector (plant avirulence determinant); 35.6%)
g10075.t1	Pfam LysM (PF01476); CBM50 (dbCAN)	Non-effector	*Cercospora beticola* (effector (plant avirulence determinant); 34.4%)

LysM domains were identified from Pfam annotations (PF01476; eggNOG-mapper v2) and dbCAN CBM50 assignments; secretion was predicted with SignalP 6.0.

Table S7: Verification of the two candidate S23-specific BGC regions (NODE_27 region 1, T1PKS; NODE_2 region 6, NRPS–terpene) against the 13 reference genomes.

Genome	NODE_27 r1 core T1PKS (2,214 aa): identity % (coverage %)	NODE_27 r1 accessory genes: detected; median identity %	NODE_27 r1 accessory genes co-located on one contig; core best hit on that contig	NODE_2 r6 core NRPS (2,841 aa): identity % (coverage %)	NODE_2 r6 accessory genes: detected; median identity %
A. tubingensis HFAT15	31 (84)	22/22; 95	22/22; no	75 (97)	21/21; 82
A. tubingensis JS3-R1	31 (84)	22/22; 96	22/22; no	42 (66)	20/21; 88
A. tubingensis WU-2223L	31 (83)	22/22; 93	22/22; no	40 (66)	20/21; 79
A. tubingensis CBS 134.48	31 (84)	22/22; 95	22/22; no	81 (99)	20/21; 74
A. tubingensis C2-2	29 (83)	22/22; 94	22/22; no	41 (66)	20/21; 88
A. luchuensis IFO 4308	31 (83)	22/22; 92	22/22; no	75 (77)	20/21; 77
A. piperis CBS 112811	31 (83)	22/22; 93	22/22; no	75 (99)	20/21; 77
A. neoniger CBS 115656	29 (98)	22/22; 94	22/22; no	80 (99)	21/21; 77
A. eucalypticola CBS 122712	31 (83)	22/22; 93	21/22; no	42 (66)	20/21; 83
A. niger CBS 513.88	31 (84)	22/22; 85	22/22; no	65 (66)	20/21; 64
A. welwitschiae CBS 139.54	39 (41)	22/22; 86	18/22; no	50 (69)	20/21; 75
A. brasiliensis CBS 101740	38 (42)	22/22; 83	20/22; no	42 (66)	21/21; 73
A. carbonarius ITEM 5010	39 (41)	21/22; 71	14/22; no	43 (67)	20/21; 59

Core enzymes: best BLASTP hit in each proteome (E < 1e-10). Accessory genes: all other proteins encoded in the region, searched by tBLASTn against the raw genome sequence (E < 1e-10). A core-enzyme identity ≥ 70% was taken as evidence of an ortholog. Co-location: number of NODE_27 r1 accessory genes whose best tBLASTn hit lies on the same contig of the panel genome; “no” indicates that the best core-PKS hit lies on a different contig, i.e., the corresponding locus lacks the polyketide synthase.

Table S8: Genome-level (tBLASTn) and protein-level (BLASTP) detection of the ten yanuthone D cluster genes across the 14 genomes panel.

Genome	*yanR*	*yanC*	*yanB*	*yanG*	*yanA*	*yanH*	*yanI*	*yanD*	*yanE*	*yanF*
A. tubingensis S23	80/98 (82)	94/97 (96)	92/95 (94)	90/94 (89)	93/100 (93)	60/100 (97)	78/100 (80)	90/100 (91)	87/100 (94)	74/95 (83)
A. tubingensis HFAT15	75/96 (81)	87/97 (88)	–	–	–	–	–	92/100 (89)	–	–
A. tubingensis JS3-R1	76/96 (85)	89/96 (87)	–	–	–	–	–	92/100 (93)	–	–
A. tubingensis WU-2223L	76/96 (63)	88/97 (87)	–	–	–	–	–	92/100 (93)	–	–
A. tubingensis CBS 134.48	75/96 (80)	87/97 (90)	–	–	–	–	–	92/100 (92)	–	–
A. tubingensis C2-2	74/96 (77)	78/97 (90)	–	–	–	–	–	92/100 (93)	–	–
A. luchuensis IFO 4308	72/96 (59)	74/43 (84)	–	–	–	–	–	92/100 (92)	–	–
A. piperis CBS 112811	71/96 (58)	80/39 (72)	–	–	–	–	–	92/100 (92)	–	–
A. neoniger CBS 115656	72/96 (73)	66/94 (78)	–	–	–	–	–	92/100 (92)	–	–
A. eucalypticola CBS 122712	–	–	–	–	–	–	–	–	–	–
A. niger CBS 513.88	90/98 (85)	99/96 (99)	99/95 (99)	99/94 (100)	99/100 (99)	74/100 (98)	84/100 (84)	93/100 (98)	98/100 (98)	73/95 (87)
A. welwitschiae CBS 139.54	90/98 (85)	99/96 (100)	100/95 (100)	99/93 (99)	99/100 (99)	59/97 (98)	84/100 (83)	92/100 (98)	98/100 (99)	76/95 (86)
A. brasiliensis CBS 101740	75/98 (69)	85/100 (95)	80/97 (85)	91/89 (91)	87/100 (87)	68/100 (95)	77/100 (77)	88/100 (89)	80/100 (91)	69/92 (83)
A. carbonarius ITEM 5010	56/83 (64)	65/89 (63) E	–	–	–	–	–	67/90 (65)	–	–

Values: tBLASTn identity (%)/query coverage (%) of the *A. niger* ATCC 1015 reference protein (UniProt G3Y415–G3Y424) against the raw genome sequence, with BLASTP identity (%) of the corresponding annotated protein in parentheses. A gene is scored as present when its best hit is co-located with the other cluster genes and the higher of the two identities is ≥60%. E, homolog (≥60%) located elsewhere in the genome; –, not detected (best hit <60%, paralogs only). In S23, *yanB* and *yanC* are encoded within one AUGUSTUS gene model (g4003.t1), and *yanI* corresponds to g3999.t1.

Table S9: Genome-wide tBLASTn search results for the yanuthone D core polyketide synthase (query: S23 g4001.t1, 1,788 aa) against the nine genomes lacking the complete cluster.

Genome	Best identity	Alignment length	Query coverage	E-value	Bitscore
A. tubingensis CBS 134.48	28.7%	1,096 aa	63%	1.61e-102	368
A. tubingensis WU-2223L	30.1%	1,046 aa	75%	1.98e-103	371
A. tubingensis C2-2	29.4%	1,091 aa	64%	1.80e-109	390
A. tubingensis JS3-R1	27.9%	1,035 aa	65%	8.61e-96	346
A. tubingensis HFAT15	28.9%	1,028 aa	82%	1.56e-109	390
A. luchuensis	30.4%	1,040 aa	60%	5.68e-102	366
A. neoniger	29.4%	1,055 aa	64%	1.16e-89	327
A. piperis	27.9%	1,082 aa	64%	1.10e-73	275
A. eucalypticola	29.8%	1,042 aa	61%	1.28e-92	336

Table S10: Amino-acid identity of S23 genes g3985–g4015 (yanuthone D locus and flanks) to their best hits in *A. tubingensis* HFAT15, *A. tubingensis* CBS 134.48 and *A. niger* CBS 513.88.

S23 gene	Position / cluster gene	*A. tubingensis* HFAT15	*A. tubingensis* CBS 134.48	*A. niger* CBS 513.88
g3985.t1	upstream flank	96 (100)	96 (100)	81 (100)
g3986.t1	upstream flank	100 (100)	100 (100)	94 (100)
g3987.t1	upstream flank	98 (100)	99 (100)	95 (100)
g3988.t1	upstream flank	97 (100)	96 (100)	89 (100)
g3989.t1	upstream flank	100 (100)	100 (100)	94 (100)
g3990.t1	upstream flank	99 (100)	99 (99)	96 (99)
g3991.t1	upstream flank	98 (89)	96 (100)	79 (97)
g3992.t1	upstream flank	100 (100)	100 (100)	98 (99)
g3993.t1	upstream flank	100 (69)	100 (69)	88 (69)
g3994.t1	upstream flank	99 (100)	96 (59)	–
g3995.t1	upstream flank	100 (100)	100 (100)	89 (100)
g3996.t1	*yanF*	30 (98)	30 (99)	90 (99)
g3997.t1	*yanE*	39 (53)	41 (74)	94 (100)
g3998.t1	*yanD*	86 (98)	90 (98)	91 (100)
g3999.t1	*yanI*	28 (62)	27 (70)	82 (99)
g4000.t1	*yanH*	32 (97)	32 (97)	97 (100)
g4001.t1	*yanA*	33 (54)	32 (56)	93 (100)
g4002.t1	*yanG*	43 (91)	45 (91)	95 (62)
g4003.t1	*yanB+yanC* (merged model)	91 (60)	91 (61)	95 (63)
g4004.t1	*yanR*	79 (39)	74 (97)	84 (100)
g4005.t1	downstream flank	90 (99)	90 (99)	71 (99)
g4006.t1	downstream flank	92 (69)	87 (102)	30 (39)
g4007.t1	downstream flank	89 (100)	89 (100)	30 (77)
g4008.t1	downstream flank	96 (100)	96 (100)	–
g4009.t1	downstream flank	35 (45)	97 (57)	30 (52)
g4010.t1	downstream flank	98 (100)	98 (100)	91 (100)
g4011.t1	downstream flank	96 (100)	96 (100)	44 (68)
g4012.t1	downstream flank	44 (28)	98 (80)	48 (101)
g4013.t1	downstream flank	99 (100)	99 (100)	–
g4014.t1	downstream flank	41 (30)	41 (28)	41 (30)
g4015.t1	downstream flank	47 (83)	46 (83)	99 (100)
Genome-wide median	(every 30th S23 protein)	99.7	99.2	94.8

Values: BLASTP identity (%) with query coverage (%) in parentheses. Low values for cluster genes in *A. tubingensis* reflect matches to paralogs, because these genes are absent from the partial locus. The last row gives the median identity of best BLASTP hits (≥90% query coverage) for a systematic subsample of the S23 proteome (i.e., every 30th predicted protein; n = 358).

Table S11: Comparative identity of both components of the nidulanin A pathway across the 14-genome panel. Local cluster values (ABC transporter, NRPS core, MFS transporter) are percent identity to the MIBiG reference (BGC0001699); NptA values are percent identity to the prenyltransferase NptA reference (*A. nidulans* FGSC A4, gene AN11080; UniProt C8VEJ5), following genome-wide search and phylogenetic confirmation of orthology.

Genome	ABC transporter	NRPS (core)	MFS transporter	NptA (unlinked)
S23 (this study)	64%	52%	79%	32.9%
A. tubingensis CBS 134.48	64%	52%	80%	32.4%
A. tubingensis WU-2223L	64%	52%	80%	31.6%
A. tubingensis C2-2	63%	52%	80%	32.6%
A. tubingensis JS3-R1	61%	52%	78%	31.6%
A. tubingensis HFAT15	61%	52%	79%	31.6%
A. luchuensis	64%	52%	80%	31.8%
A. neoniger	64%	52%	80%	32.6%
A. piperis	64%	52%	82%	32.8%
A. eucalypticola	63%	52%	80%	32.7%
A. niger	64%	52%	81%	32.2%
A. welwitschiae	64%	52%	81%	32.4%
A. brasiliensis	64%	52%	81%	31.4%
A. carbonarius (outgroup)	64%	53%	81%	32.2%

Supplementary Figures

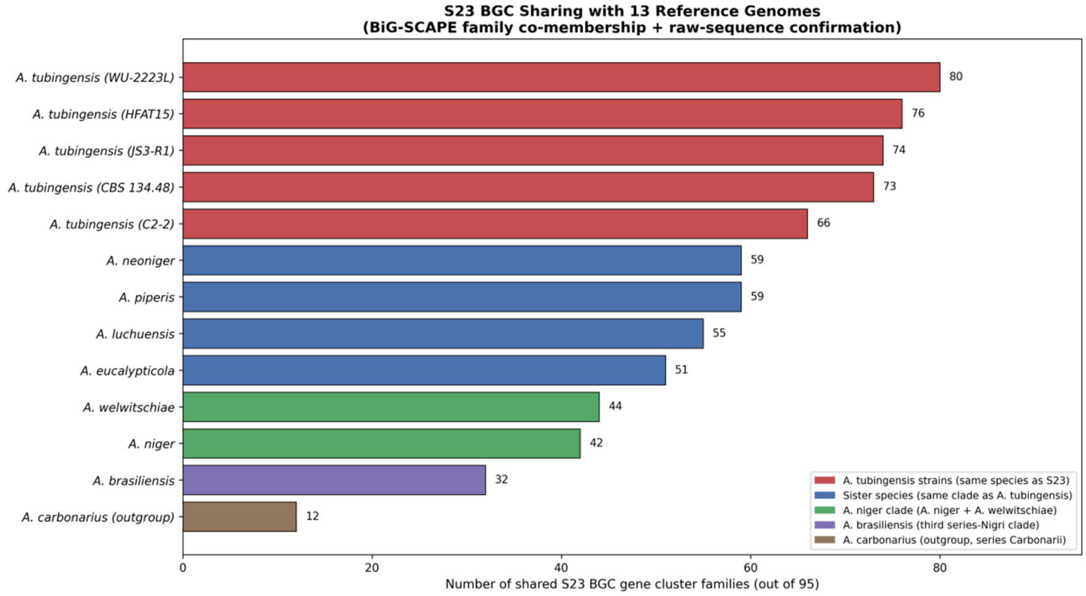

Figure S1: Percentage of isolate S23's 95 biosynthetic gene clusters sharing a BiG-SCAPE gene cluster family with each of 13 reference genomes, ordered by phylogenetic relationship to S23.

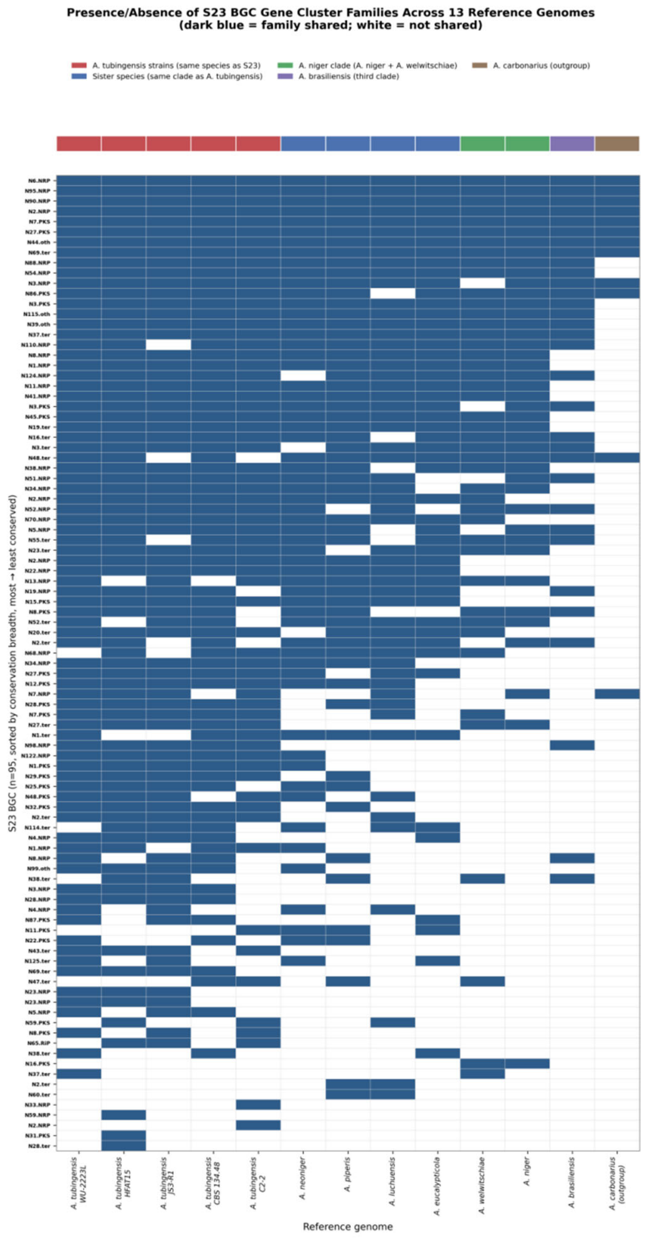

Figure S2: Presence/absence of isolate S23's 95 BGC gene cluster families across the 13 genome reference panel.

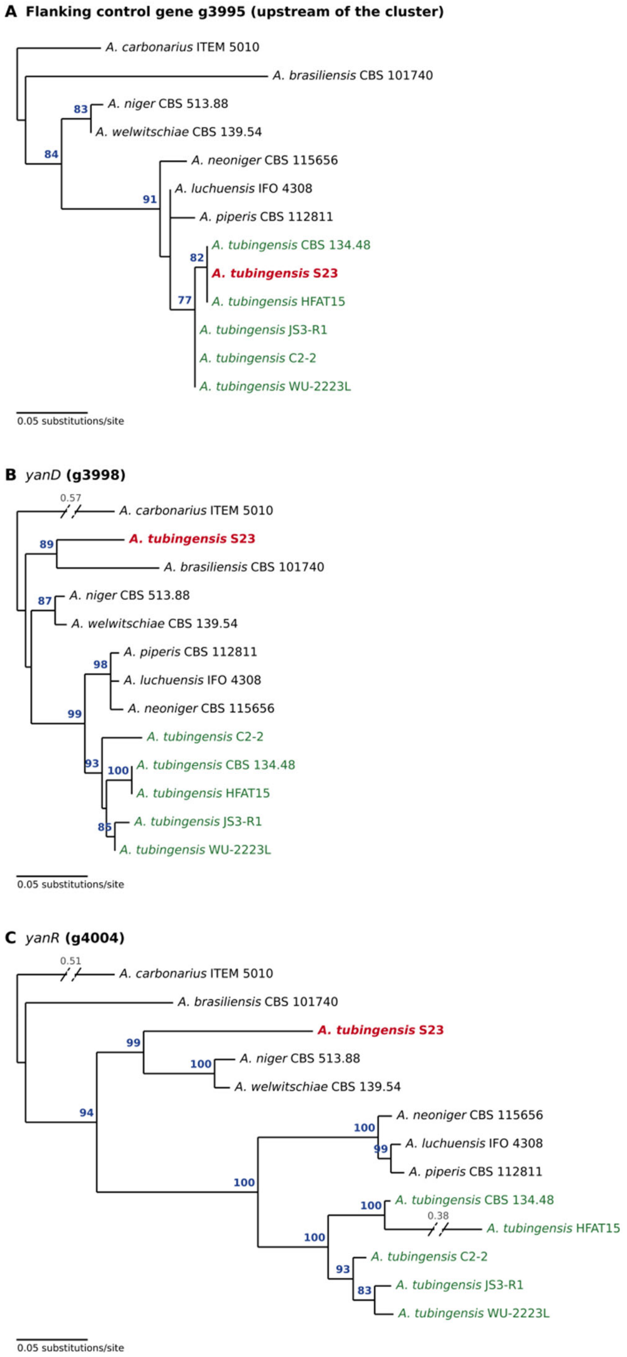

Figure S3. Maximum-likelihood gene trees for the yanuthone D locus: (A) the upstream flanking gene g3995, (B) yanD (g3998) and (C) yanR (g4004), based on the best BLASTP hit in each proteome (IQ-TREE v3.1.3, best-fit models WAG, Q.PLANT+G4 and Q.MAMMAL+G4, respectively with 1,000 ultrafast bootstrap replicates (only values ≥70 shown). Trees are rooted with A. carbonarius as the outgroup and drawn to the same scale (scale bar, 0.05 substitutions/site). Branches longer than 0.25 substitutions/site are shortened (//), with their lengths shown above the break. A. eucalypticola was excluded because it lacks the yanuthone D cluster genes. S23 is shown in red and other A. tubingensis strains in green. Alignments and tree files are provided in Supplementary Data 14.

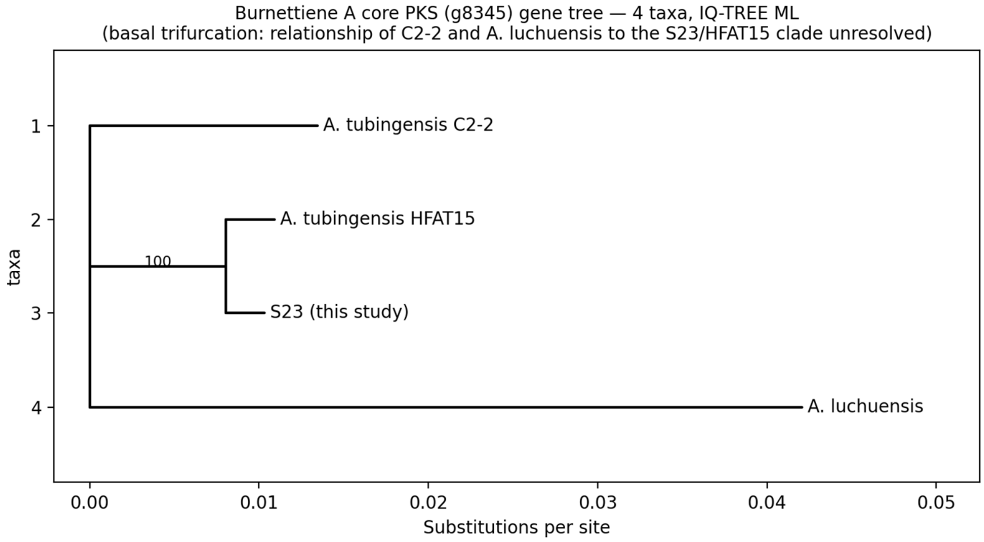

Figure S4: Maximum-likelihood gene tree of the burnettiene A core polyketide synthase (g8345) across the four genomes carrying the complete cluster (IQ-TREE2, LG substitution model, 1000 ultrafast bootstrap replicates). Branch lengths are in substitutions per site.

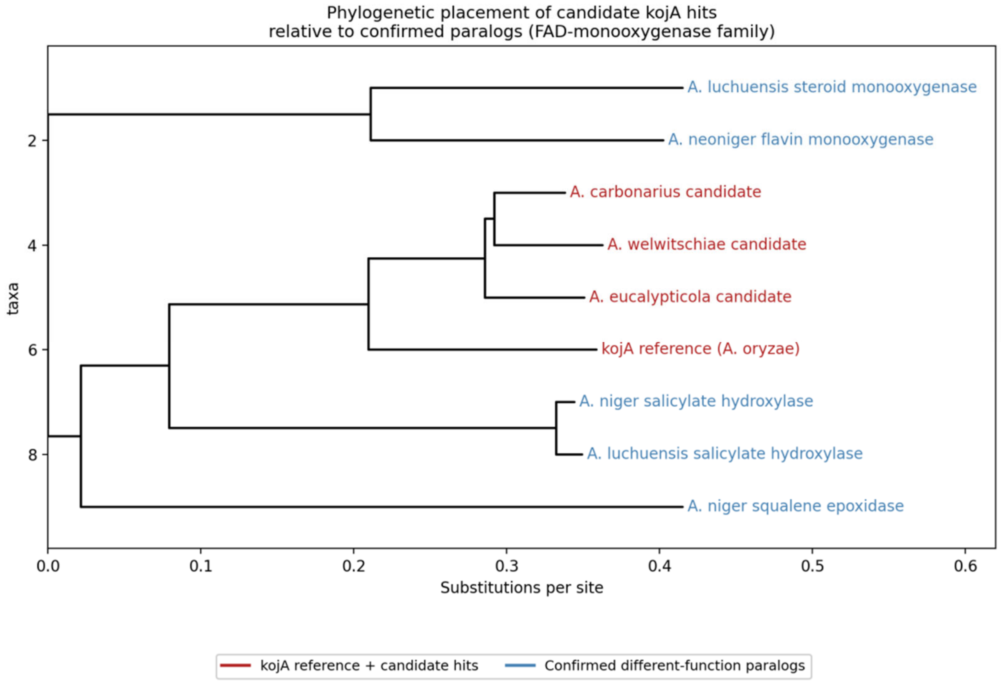

Figure S5: Neighbor-joining tree placing the three candidate kojA hits (*A. eucalypticola*, *A. welwitschiae*, *A. carbonarius*; red) alongside the true kojA reference (*A. oryzae*) and five confirmed paralogs of distinct function, i.e., salicylate hydroxylase, squalene epoxidase, and steroid/flavin monooxygenase (blue). All three candidates cluster with the kojA reference in a single, well-separated clade, distinct from the paralog clade, confirming they are genuine kojA orthologs rather than more distant family members.

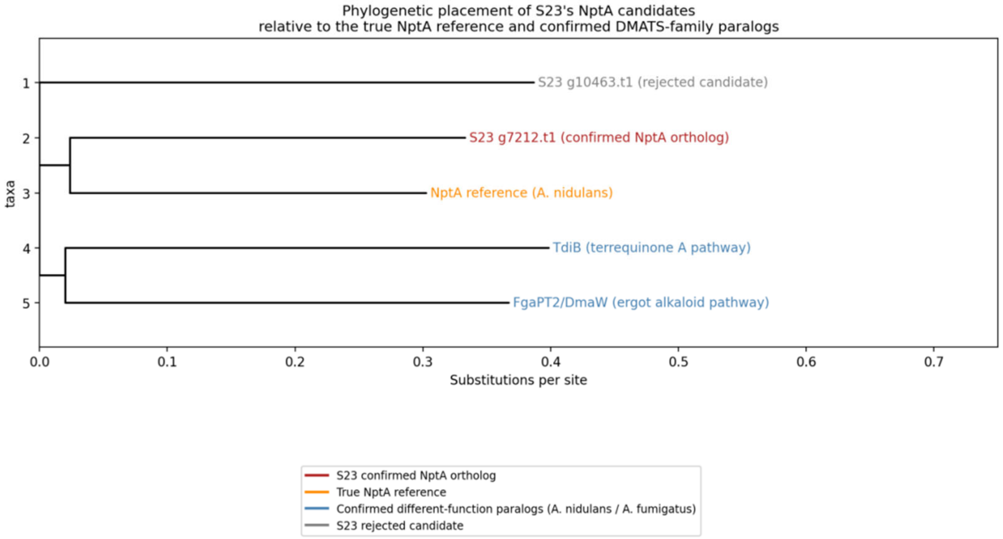

Figure S6: Neighbor-joining tree placing S23's two candidate NptA hits (g7212.t1, g10463.t1) alongside the NptA reference (*A. nidulans*) and two confirmed, functionally distinct prenyltransferase-family paralogs (TdiB, terrequinone A pathway; FgaPT2/DmaW, ergot alkaloid pathway). g7212.t1 clusters specifically and tightly with the NptA reference (41.2% pairwise identity), confirming its genuine orthology. The g10463.t1 falls as an outgroup to all reference sequences, excluding it as a candidate.

Supplementary Data

Supplementary Data 1–32 are provided as separate files in the archive S23_Supplementary_Data_R1.zip (file names begin with Supplementary_Data_01 … Supplementary_Data_32).

No.	Description	File
Supplementary Data 1	run_dbcan v5.2.9 CAZyme annotation of the S23 proteome (high-confidence calls = supported by >=2 of 3 methods)	Supplementary_Data_01_dbCAN_CAZyme_overview.tsv
Supplementary Data 2	SignalP 6.0 predictions for the 919 secreted S23 proteins with EffectorP-fungi 3.0 classification	Supplementary_Data_02_SignalP6_EffectorP3_secretome.tsv
Supplementary Data 3	BLASTP matches of the S23 secretome to PHI-base with host, disease and mutant phenotype	Supplementary_Data_03_PHIbase_secretome_hits.csv
Supplementary Data 4	All 95 antiSMASH BGC regions of S23 with product types, coordinates and KnownClusterBlast top hit	Supplementary_Data_04_S23_all_95_BGCs.xlsx
Supplementary Data 5	KnownClusterBlast (MIBiG) hits of S23 BGC regions	Supplementary_Data_05_antiSMASH_KnownClusterBlast_hits.csv
Supplementary Data 6	BiG-SCAPE gene cluster family assignments	Supplementary_Data_06_BiGSCAPE_GCF_assignments.csv
Supplementary Data 7	GCF sharing between S23 and each reference genome (full table)	Supplementary_Data_07_BGC_sharing_full_table.csv
Supplementary Data 8	GCF sharing summary and patristic distances	Supplementary_Data_08_BGC_sharing_summary_table.csv
Supplementary Data 9	S23 yanuthone D cluster protein sequences (g3996.t1-g4004.t1)	Supplementary_Data_09_yanuthoneD_S23_query_sequences.faa
Supplementary Data 10	Protein-level searches of S23 cluster genes against the reference genomes	Supplementary_Data_10_yanuthoneD_identity_standard_threshold.tsv
Supplementary Data 11	S23 yanuthone D polyketide synthase (YanA, g4001.t1) query sequence	Supplementary_Data_11_yanuthoneD_PKS_query_sequence.faa
Supplementary Data 12	Genome-wide tBLASTn search for YanA in genomes lacking the complete cluster	Supplementary_Data_12_yanuthoneD_PKS_genomewide_search_summary.csv
Supplementary Data 13	Query coverage matrix for the yanuthone D cluster genes across the panel	Supplementary_Data_13_yanuthoneD_coverage_matrix.tsv
Supplementary Data 14	MAFFT alignments and IQ-TREE ML trees for g3995, yanD (g3998) and yanR (g4004) (Figure S3)	Supplementary_Data_14_yanuthoneD_locus_gene_tree_alignments_and_trees.txt
Supplementary Data 15	S23 burnettiene A cluster protein sequences (g8345.t1-g8352.t1)	Supplementary_Data_15_burnettieneA_S23_query_sequences.faa
Supplementary Data 16	tBLASTn identity of burnettiene A cluster genes across the panel	Supplementary_Data_16_burnettieneA_identity_all_genomes.tsv
Supplementary Data 17	Presence classification of burnettiene A cluster genes across the panel	Supplementary_Data_17_burnettieneA_classification_matrix.tsv
Supplementary Data 18	Core PKS (g8345) sequences used for the burnettiene A gene tree	Supplementary_Data_18_burnettieneA_g8345_tree_sequences.faa
Supplementary Data 19	IQ-TREE maximum-likelihood tree of the burnettiene A core PKS (Figure S4)	Supplementary_Data_19_burnettieneA_g8345_IQTREE_MLtree.nwk
Supplementary Data 20	IQ-TREE report for the burnettiene A core PKS tree	Supplementary_Data_20_burnettieneA_g8345_IQTREE_report.txt
Supplementary Data 21	Identity of kojR and kojT homologs across the panel	Supplementary_Data_21_kojRT_identity_all_genomes.tsv
Supplementary Data 22	Genome-wide search for kojA (UniProt Q2U5I0) in all 14 genomes	Supplementary_Data_22_kojA_genomewide_search_summary.txt
Supplementary Data 23	kojA reference sequence (*A. oryzae* RIB40, UniProt Q2U5I0)	Supplementary_Data_23_kojA_reference_sequence.faa

No.	Description	File
Supplementary Data 24	kojA candidate orthologs and paralogs of known function used for the kojA tree (Figure S5)	Supplementary_Data_24_kojA_candidates_and_paralogs.faa
Supplementary Data 25	NptA reference sequence (*A. nidulans* FGSC A4 AN11080, UniProt C8VEJ5)	Supplementary_Data_25_nidulaninA_NptA_reference_sequence.faa
Supplementary Data 26	BLASTP hits of NptA in the S23 proteome	Supplementary_Data_26_nidulaninA_NptA_S23_proteome_hits.tsv
Supplementary Data 27	Genome-wide NptA search across the 13 reference genomes	Supplementary_Data_27_nidulaninA_NptA_genomewide_search_13genomes.tsv
Supplementary Data 28	Sequences used for the NptA candidate tree (Figure S6)	Supplementary_Data_28_nidulaninA_NptA_tree_sequences.faa
Supplementary Data 29	NptA candidate tree (Newick)	Supplementary_Data_29_nidulaninA_NptA_tree.nwk
Supplementary Data 30	Structure data used to draw nidulanin A (Figure 9B)	Supplementary_Data_30_nidulaninA_structure_data.txt
Supplementary Data 31	S23 TAN-1612 cluster protein sequences (g1713.t1-g1717.t1)	Supplementary_Data_31_TAN1612_S23_query_sequences.faa
Supplementary Data 32	Identity of TAN-1612 cluster genes across the panel	Supplementary_Data_32_TAN1612_identity_all_genomes.tsv
